# Bridge-Induced Translocation between *NUP145* and *TOP2* Yeast Genes Models the Genetic Fusion between the Human Orthologs Associated With Acute Myeloid Leukemia

**DOI:** 10.3389/fonc.2017.00231

**Published:** 2017-09-29

**Authors:** Valentina Tosato, Nicole West, Jan Zrimec, Dmitri V. Nikitin, Giannino Del Sal, Roberto Marano, Michael Breitenbach, Carlo V. Bruschi

**Affiliations:** ^1^Ulisse Biomed S.r.l., AREA Science Park, Trieste, Italy; ^2^Faculty of Health Sciences, University of Primorska, Izola, Slovenia; ^3^Yeast Molecular Genetics, ICGEB, AREA Science Park, Trieste, Italy; ^4^Clinical Pathology, Hospital Maggiore, Trieste, Italy; ^5^Biology Faculty, M.V. Lomonosov Moscow State University, Moscow, Russia; ^6^Department of Life Sciences, University of Trieste, Trieste, Italy; ^7^Genetics Division, Department of Cell Biology, University of Salzburg, Salzburg, Austria

**Keywords:** acute myeloid leukemia, bridge-induced translocation, gene fusion, yeast, nucleoporin, P53

## Abstract

In mammalian organisms liquid tumors such as acute myeloid leukemia (AML) are related to spontaneous chromosomal translocations ensuing in gene fusions. We previously developed a system named bridge-induced translocation (BIT) that allows linking together two different chromosomes exploiting the strong endogenous homologous recombination system of the yeast *Saccharomyces cerevisiae*. The BIT system generates a heterogeneous population of cells with different aneuploidies and severe aberrant phenotypes reminiscent of a cancerogenic transformation. In this work, thanks to a complex pop-out methodology of the marker used for the selection of translocants, we succeeded by BIT technology to precisely reproduce in yeast the peculiar chromosome translocation that has been associated with AML, characterized by the fusion between the human genes *NUP98* and *TOP2B*. To shed light on the origin of the DNA fragility within *NUP98*, an extensive analysis of the curvature, bending, thermostability, and B-Z transition aptitude of the breakpoint region of *NUP98* and of its yeast ortholog *NUP145* has been performed. On this basis, a DNA cassette carrying homologous tails to the two genes was amplified by PCR and allowed the targeted fusion between *NUP145* and *TOP2*, leading to reproduce the chimeric transcript in a diploid strain of *S. cerevisiae*. The resulting translocated yeast obtained through BIT appears characterized by abnormal spherical bodies of nearly 500 nm of diameter, absence of external membrane and defined cytoplasmic localization. Since Nup98 is a well-known regulator of the post-transcriptional modification of P53 target genes, and *P53* mutations are occasionally reported in AML, this translocant yeast strain can be used as a model to test the constitutive expression of human *P53*. Although the abnormal phenotype of the translocant yeast was never rescued by its expression, an exogenous *P53* was recognized to confer increased vitality to the translocants, in spite of its usual and well-documented toxicity to wild-type yeast strains. These results obtained in yeast could provide new grounds for the interpretation of past observations made in leukemic patients indicating a possible involvement of *P53* in cell transformation toward AML.

## Introduction

Nucleoporins have important roles in many cellular pathways such as the nucleocytoplasmic transport (1), mitotic spindle assembly checkpoint (2), and chromatin metabolism (3). The sporadic rearrangement of their encoding genes lead to aberrant chimeric proteins often implicated in hematologic malignancies such as the acute myeloid leukemia (AML) (4). In particular, the amino terminus of Nup98 is known to be involved in the fusions with at least 28 different partners provoking different types of leukemia (5). In the past, several mouse models of retroviral-introduced artificial fusions have been developed (6, 7) and potential therapeutic targets for specific Nup98 fusions-mediated transformation are under studies (8), but the exact role of the Nup98 chimeras in cell immortalization remains still unclear (9, 10). Moreover, previous works were mostly focused on the clinical effects of the chimeric fusion rather than on the genetic etiology of the disease. We, therefore, planned an experiment to verify whether a chromosomal translocation involving *NUP98* might be reproduced in a model organism such as *Saccharomyces cerevisiae* using our previously published bridge-induced translocation (BIT) system (11, 12). Among all the different possible chimeras leading to hematopoietic malignancies, we focused on a peptide resulting from the fusion between the genes *NUP98* and *TOP2B* that had been found in a patient with primary AML (13). The choice of these two loci required a substantial long period of time and was dictated (i) by the necessity of a detailed sequence description of a translocation breakpoint in human cells, (ii) by the fact that these two genes (*NUP98* and *TOP2B*) have orthologs in yeast and, (iii) by the topological orientation of these orthologs allowing the formation of a viable translocated yeast cell. The patient described in this paper (13) achieved complete histologic remission but relapsed 15 months after diagnosis and showed a 90% blast cell infiltration in the bone marrow. The blast cells were resistant to a combination of Ara-C and topotecan, usually efficient in cases without a complete response (14), with a negative outcome (13). In this work, the precise DNA junction between the two genes had been clearly described, providing partial sequences of the *NUP98/TOP2B* fusion. The breakpoint within *NUP98* (Chromosome 11) occurred at nucleotide 1,199 of the intron 13 with a consequent deletion of two base pairs, while the breakpoint of *TOP2B* (Chromosome 3) occurred within nucleotide 687 within intron 25, with a duplication of four base pairs (13). An important point is that at least two different reciprocal chimeric transcripts (*TOP2B–NUP98*) were identified, suggesting that a precise reciprocal construct is not essential for the leukemic transformation process. This observation is confirmed by other analyses of *NUP98* fusions, where the reciprocal transcript was never found in leukemic patients (15, 16). Since BIT produces always non-reciprocal chromosome translocations (17, 18), the demonstration that the reciprocal transcript(s) did not play a role in the oncogenic transformation reinforced the idea to use BIT in the modeling of this particular translocation. BIT allows to precisely link together through a linear DNA cassette two targeted *loci* on different chromosomes, exploiting the Rad52/Rad54-dependent endogenous homologous recombination system (HRS) of the yeast cell (11, 18). The efficiency of the resulting translocation event depends on the length of the homologous ends, on the selected yeast strain and on the DNA stability of the targeted *loci* (19). Using BIT followed by a pioneering pop-out technology we succeeded to generate an *in vivo* perfect fusion between the genes *NUP145* (ortholog to human *NUP98*) and *TOP2* (ortholog to human *TOP2B*). Extensive conformational and physicochemical analyses of the DNA region around the breakpoints detected similarities and differences between yeast and mammalian DNA and enlightened the basis of the DNA weakness of *NUP98*. BIT produced a peculiar NUP-TOP translocated yeast strain (TNT), suggestively resembling a “leukemic yeast,” which can be easily manipulated to investigate the genetic origin of the leukemic translocation process and the molecular players involved. Aged TNT cells are phenotypically characterized by huge, abnormal, spherical bodies (SBs), which can be stained by the RNA-intercalator Pyronin Y (PY). Differently from other types of tumors, *P53* is rarely mutated in hematological malignancies while it is vice versa recurrently overexpressed (20–22). After constitutive expression of human *P53* in TNT, in spite of the lack of a phenotypic reversion, an increased vitality and vigorous growth were observed. These data were completely unexpected since constitutively expressed *P53* was found to be toxic in the parental wild-type (WT) yeast strain, in agreement with previous observations (23). Therefore, these results obtained in the model translocant yeast indicate that the human P53 is an energy booster for aneuploid yeast cells and, corroborating previous clinical data, suggest that P53 might be an indicator of cell transformation to AML (24, 25) and its presence predictive of adverse prognosis (22). Moreover, we propose that the unusual phenotype of aged TNTs, characterized by huge RNA-rich SBs, could be related to the translocation involving Nup145. Further insights on the role of this nucleoporin in the function, size, and integrity of cytoplasmatic bodies are currently under investigation.

## Materials and Methods

### Strains and Media

The diploid strain San1, constructed in our laboratory (11, 26) by mating Fas20 (α, ade1 ade2 ade8 can1R leu2 trp1, ura3-52) (26) and YPH250 (a, ade2-101^o^ leu2-Δ1 lys2-801a his3-Δ200 trp1-Δ1 ura3-52, ATCC 96519), was used to obtain the TNTs and as control strain throughout this work. To plot the growth curves, the cells were counted every 2 h and the values expressed in 10^7^ cells/ml. Each value is the result of three independent readings and its error bar is reported accordingly.

Yeast peptone dextrose (YPD), supplemented with geneticin (G418, final conc. 200 µg/ml, Gibco), and Synthetic Complete (SC)—URA were used as selective media. To select for the TNTs, the SE drop-out-medium (with ammonium glutamate instead of ammonium sulfate) was prepared as previously described (27). 5-Fluoroorotic acid (5-FOA) plates were prepared optimizing the protocol from Akada (28) with an increased amount of 5-FOA up to 1.2 g/l and a decreased amount of uracil (final concentration ≤20 mg/l) to minimize background.

### Translocants Construction and Analysis

Plasmid pFA6aKlura (29) was used to amplify the gene *URA3* from *Kluyveromyces lactis* (KlURA). while the plasmid pFA6aKANMX4 (30) was used as template to amplify the kanamycin gene. URA prototroph and G418-resistant transformants were obtained using the lithium-acetate transformation for the PCR-based gene replacement method (30). To obtain different constructs we prepared a template with 320 bp of *NUP145* that were cut PstI/BamHI and cloned upstream the *KlURA* gene on the pFA6aKlura plasmid while 150 bp of *NUP145* were cut SacI/EcoRI and cloned downstream the same plasmid as shown in Figure S1D in Supplementary Material. All the primers used to amplify the constructs and to verify them are listed in Table S1 in Supplementary Material. The constructs were all amplified by High-Fidelity PCR (Kapa Biosystems), purified and verified by sequencing (BMR sequencing service, Padova). The total amount of cells per transformation was 2.2 × 10^8^ and the efficiency (E) of each transformation was determined dividing the frequency (ν) for the DNA amount in microgram used in the transformation process (ν/μg DNA) (19).

Chromosome separation by contour-clamped homogeneous electric field (CHEF) and Southern hybridization were performed as previously reported (11) using probes amplified with primers listed in Table S1 in Supplementary Material. For the Gene Copy Number by quantitative PCR, the DNA was extracted using the Wizard Genomic DNA purification kit (Promega), then it was diluted from 10 to 50 ng/µl and quantified with a GeneQuant Pro spectrophotometer (NanoDrop1000, Thermo scientific) in order to define a standard curve, using as reference genes *ACT1* on chromosome VI and *SSE2* on chromosome II (31). Copy number of unknown samples was calculated from the standard curve by using the equation copy number = 10^(Ct − ^*^b^*^)/^*^m^*, where *b* and *m* represents *y* intercept and slope, respectively.

The DNA copy number and the RT-PCR were run in a Rotor-GENE Q PCR (Qiagen) using the Rotor-Gene SYBR green KIT (Qiagen) and standard programs recommended by the supplier.

The data analysis of the RT-PCR was performed repeating the experiments at least three times and the relative gene expression (RGE) was calculated with the comparative C (T) method (32) using *ACT1* as internal control gene and either the WT San1 or the translocant (both transformed with the empty pJL49) as reference strain to calculate the ΔC (T) values.

### POP-OUT Selection of the Translocants and Stability Checking

The translocants carrying the *KlURA* marker and labeled with the *KAN* gene on the translocated chromosome were grown for 2 days in non-selective medium and then overnight, from a fresh inoculum, in YPD + G418. The cells were plated on 5-FOA in serial dilutions from 3.2 × 10^7^/plate to 1 × 10^5^/plate using as positive control the auxotrophic strain San1 and as negative control a prototrophic WT yeast strain. After replica plating to eliminate the background, the putative POP-OUT clones were re-streaked on SC-URA and on 5-FOA and verified by Southern blot, colony PCR, and genomic-PCR. In the case of a positive POP-OUT response (with consequent elimination of the marker), a DNA stretch (named “scar” because it was the result of homologous recombination even if it did not contain any exogenous DNA) of 659 bp was amplified with primers FwNUP and REVTOP (Table S1 in Supplementary Material) and sequenced. In all the figures, if not differently specified, the standard DNA ladder was always the 1 kb Plus (Invitrogen). Stability tests of the translocated chromosome were performed growing each TNT translocant in non-selective medium, plating 100 µl of a 2 × 10^3^/ml dilution on 40 YPD plates and replicating them on G418 after 2 days.

### Microscopy

DAPI and *FUN-1* staining (Molecular Probes, OR, USA) were performed as previously described (17) using a Leica DMBL photomicroscope equipped with a CCD computer-driven camera at 60× and 100× magnifications. The endocytosis assay was performed using the dye lucifer yellow (LY, Sigma; final conc. 4 mg/ml) with an optimized Riezman protocol (33), as previously described (34), and detecting the fluorescence in the FITC channel. The PY (Sigma) staining on yeast cells was performed dissolving the powder in an acid solution (15% acetic acid in water) and diluting it several times with water, reaching 1 µg/ml as optimal working concentration. The yeast cells were then centrifuged, washed twice with water (to eliminate the background generated by the medium), and resuspended in a maximum of 50–100 ng/ml PY solution. The amount of PY was never exceeded to avoid signal interference from the nuclear DNA, as also previously suggested at the FOM2011 Conference by Rybak (35).^1^ When cell wall visualization was needed, Calcofluor White M2R (final concentration 25 µM) was added to the yeast cell suspension already labeled with PY. After incubation at 30°C in the dark for few minutes, fluorescence was detected exciting PY and Calcofluor with green and UV light, respectively.

After 4 weeks of continuous growth, the morphology of TNTs was analyzed through transmission electron microscopy (TEM). The fixation step with glutaraldehyde was followed by a postfixation step with osmium tetroxide and by a dehydration step with increasing percentages of ethanol. Then the samples were embedded within the epoxy resin Derr 332-732. After a three-day resin polymerization, the samples were cut in 10–20 nm-thick sections with an ultramicrotome (Leica Ultracut UCT) equipped with a diamond blade (Drukker). Sections were thus laid down in a holder grid and incubated 10 min at room temperature with a 0.1% (w/v) lead citrate solution and a 2% (w/v) uranyl acetate solution; after each incubation, the grid was washed 20 times by immersion in water. Finally, samples were observed with a transmission electron microscope EM 208 (Philips) equipped with a Morada 4,008 × 2,672 pixels 14 Bit-camera and an acquiring system Olympus Soft Imaging Solutions GmbH.

### Bioinformatics

Physicochemical and conformational properties of the DNA breaking strands were predicted with different models: (i) relative DNA duplex stability (*dG*) with the thermodynamic nearest-neighbor model and unified free energy parameters at 37°C (36); (ii) thermally induced duplex destabilization (TIDD) with the TIDD server^2^ (37) using the M5P model with 6 bp neighboring regions and a threshold of 0.1 Å; (iii) B-to-A (*BA*) and B-to-Z (*BZ*) transitions according to the dinucleotide model parameters described in Lisser and Margalit (38); (iv) DNA bending, complexity, and curvature analysis were performed using the bend.it® server (39) with a curvature/complexity window size of 50–70 nt and a cubic spline smoothing. Sequence complexity, calculated according to the Shannon entropy or Kolmogorov methods, had been previously defined (40). Bending was considered as produced by a rolling of adjacent base pairs over one another about their long axes with the tilting of base pairs about their short axes that could make a contribution. By contrast, curvature was defined as relatively macroscopic DNA bend, representing the intrinsic tendency of DNA to follow a non-linear pathway over an appreciable length, which is a result of variation of local bends in phase with the DNA helix (41); (v) DNA persistence length (*z*, proportional to bending rigidity) and DNA helical repeats (*h*, number of bps per helix turn) with the model based on cyclization experiments of short DNA fragments (42). The structural properties were predicted in windows of 100 bp, except with TIDD, where 20 nt was used, and the bending/curvature, where windows from 70 to 100 nt were used. To build the control range, five known fragile sequences under ongoing spontaneous breaks (three from *TEL1/ETV6*, one from *PML2*, and one from the *RARA* gene) have been analyzed with the same programs.

Sequences aligning and comparisons were verified with the NCBI database using the specialized BLAST bl2seq. Functionally, conserved aminoacids (aa) were found and represented with the Seq2Logo programme exploiting the Kullback–Leibler logo type and the Hobohm 1 clustering method, correcting the displayed frequencies for low number of observations as described (43). Regulatory DNA motifs were identified running SCOPE 2.1.0 [Dartmouth College, NH, USA; (44)] on both DNA strands.

## Results

### Identification of the Translocation Breakpoint in Yeast

**Figure 1:**
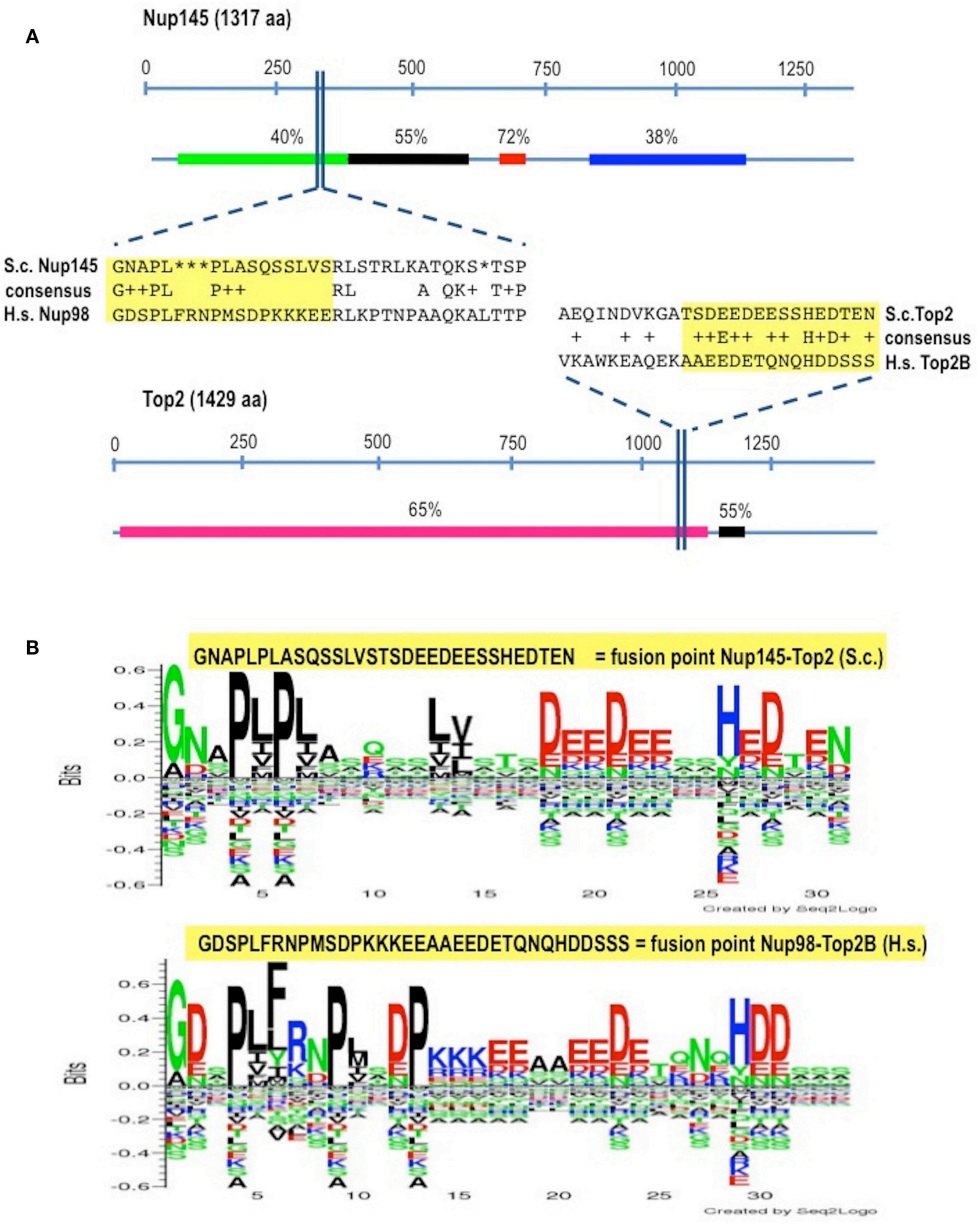
Identification of the virtual breakpoints within the yeast proteins Nup145 and Top2 through an alignment with the human orthologs. **(A)** Breakpoints within the yeast proteins and their homology in percentages with the human orthologs (line below each protein) are shown. In the windows, a partial sequence alignment between yeast (S.c.) and human (H.s.) proteins and the relative consensus are presented. The parts of the proteins that are going to be fused together resulting in chimeras are outlined in yellow. aa, amino acids. **(B)** The fusion points in yeast (top panel) and in human (bottom panel) are represented by the Seq2Logo server. Large symbols represent frequently observed amino acids, big stack represents conserved positions and small stack represents variable positions. The *Y*-axis describes the amount of information in bits. The *X*-axis shows the position in the alignment.

**Figure 2:**
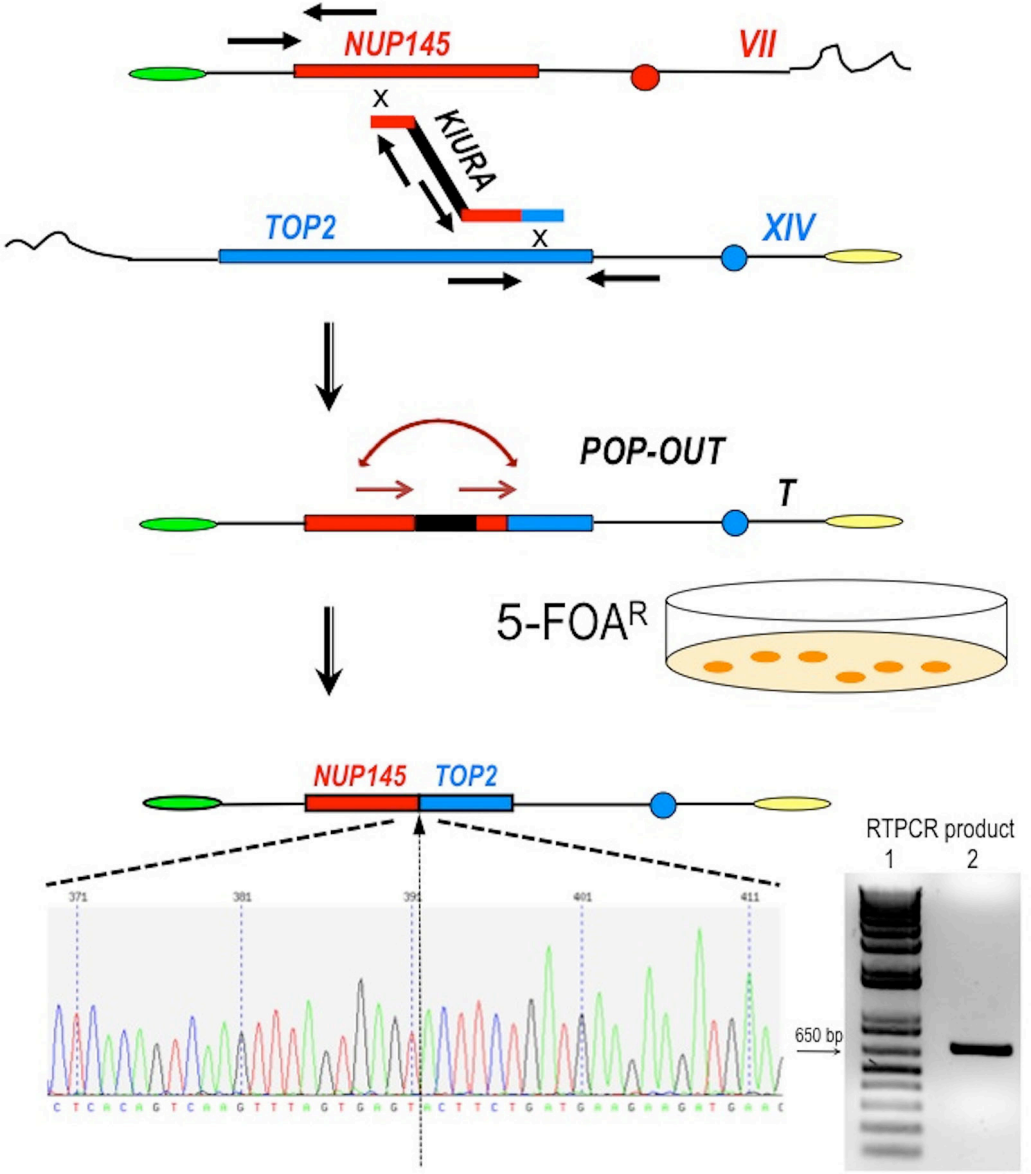
Scheme of the procedure to obtain a perfect fusion between *NUP145* and *TOP2*. The first step is represented by the bridge-induced translocation between *NUP145* (Chromosome VII, shown in red) and *TOP2* (Chromosome XIV, shown in blue) triggered by a BIT cassette. The *URA3* gene from *Kluyveromyces lactis* (KlURA) was used as selection marker between the two homologous ends. Its POP-OUT, indicated by sequential arrows, was verified by selection on 5-FOA plates. The original chromatogram of one clone showing the perfect fusion between the two genes and the relative amplified chimeric transcript from one translocant (T) are also reported. Since the primers used in the RTPCR (sequence in Table S1 in Supplementary Material) are 418 and 241 bp far from the junction point, respectively, the size of the amplicon shown here is exactly 659 bp (lane 2 of the gel); lane 1:1 kb Plus ladder (Invitrogen).

In *Homo sapiens* (*Hs*) Nup98 is coded together with Nup96 by the same open reading frame and produced by autopeptidase cleavage. In *S. cerevisae* (*Sc*) the whole length of the protein is 1,317 amino acids-long and it is composed of 605 residues of N-terminal Nup145 (ortholog to Nup98) and of 712 residues of C-terminal Nup145 (ortholog to Nup96). We found that the identity (BLASTp) between *Hs*Nup98 and *Sc*Nup145 is on average 36% with peaks of 72% within short regions while the identity between *Hs*Top2B and *Sc*Top2 is on average much higher (Figure 1A) since topoisomerases are well conserved, with structural insights from the yeast enzymes that are likely to apply to the human ones (45). The most conserved region (90% positives) between Nup98 and Nup145 is represented by a hydrophobic stretch of 11 residues immediately before the breakpoint. Exploiting the good level of homology between yeast and human sequences, it was, therefore, easy to pinpoint the corresponding, virtual breakpoint in *S. cerevisiae* (Figure 1) and to design the primers (Table S1 in Supplementary Material) for a BIT cassette amplification that would model the chromosomal translocation etiology of the leukemic transformation in yeast (Figure 2).

### Construction of the Translocation “NUP-TOP” (TNT)

To generate the translocation between *nup* and *top* loci, we implemented a methodology based on the following steps and briefly summarized in Figure 2: (a) amplification of a DNA cassette carrying the selection marker and two homologies toward *nup145* (Chromosome VII) and *top2* (Chromosome XIV) loci; (b) transformation of the yeast diploid strain San1 and selection for the correct translocants on G418 plates; (c) POP-OUT of the marker and verification of the precise fusion of the two coding sequences. *KlURA3*, the orotidine-5′-phosphate decarboxylase gene from *K. lactis*, whose loss can be easy counter-selected on 5-FOA, was chosen as selective marker (see Materials and Methods for details). We planned to perform the POP-OUT of the marker exploiting the homologous recombination between direct repeats (Figure 2). Since the recombination frequency between 40 bp repeats in yeast vegetative cultures was calculated as 2.9 × 10^−6^, with a 100-fold variation range, probably due to the intrinsic property of the selected sequence (28), we decided to test a set of constructs differing among them for the length of the repeat (from 40 to 800 bp) and for the length of the homologous ends (from 40 to 100 bp, see Table S1 in Supplementary Material). When the short repeat (40 bp) and the standard length of homology of a BIT cassette [65 bp (11, 19)] were used, the efficiency of the translocation NUP-TOP was very poor (0.6%) while when the repeats were extended up to 800 bp, even single site integration (SSI) events were favored against BIT [data not shown; for a detailed description of the differences between BIT and SSI pathways see Ref. (46)]. The optimal length of the repeat was found to be around 150 bp while the ideal length of the homologies for an efficient targeting was 100 bp. Using this optimized BIT cassette, we were able to find 6 clones, out of 177 screened, with both ends integrated in the corrected loci (Table S2 in Supplementary Material). Surprisingly, none of them gave rise to the amplification of the bridge either by colony or genomic PCR even using two different sets of primers chosen on the two chromosomes around the translocation breakpoint (Table S1 in Supplementary Material). Moreover, sequencing of the junctions showed point mutations and/or rearrangements in four out of these six clones and Southern blot hybridization revealed that only one of them (cl. 112) had the translocant of the expected size (Figure S1C in Supplementary Material). We assumed that, in the majority of clones, a faulty recombination had happened among different copies of the cassette inside the cell leading to an unwanted concatemer (Figure S1B in Supplementary Material). This hypothesis was verified by colony PCR with primers k1 and k2 (Figure S1B in Supplementary Material) and was confirmed by quantitative PCR analysis, which detected at least 10 copies of the cassette. The concatemer was also generated with short repeats of at least 40 bp (data not shown). In order to reduce the length of the concatemer and to get the correct TNT, clone 112 was left growing in rich, non-selective medium for 1 week. Then it was diluted and plated on YPD, -URA, and 5-FOA medium. Among all the clones screened, one (cl.15) that was strongly flocculating, grew on YPD and on -URA, negligibly on 5-FOA, and had the expected size (2,460 bp) of the correct bridge between *nup145* and *top2* loci. This clone was verified by sequencing and Southern blot and it was subsequently used for the POP-OUT of the marker *KlURA* (Figure 2).

Before the POP-OUT, the translocated chromosome of the TNT strain was labeled to avoid false positives on 5-FOA (clones that grow on 5-FOA because they have lost the whole translocated chromosome and not the *KlURA* marker only). We, therefore, introduced the KAN gene within the right arm of the translocated chromosome, between *PHO91* and *YNR014W* (Figure S2 in Supplementary Material—for the primers sequence and their exact location see Table S1 in Supplementary Material).

The POP-OUT was performed as described in Section “Materials and Methods” and happened with an approximate frequency of 1 × 10^−6^. The 659 bp-scar left by the *KlURA* gene was amplified with primers FwNUP and RevTOP (Table S1 and Figure S1 in Supplementary Material; Figure 2) and sequenced. The POP-OUT resulted in the wanted, perfect junction between chromosome VII (position 338787) and chromosome XIV (position 460959) (the partial sequence and chromatogram is reported in Figure 2). We repeated the procedure (KAN labeling, POP-OUT followed by replica and selection) three different times obtaining nine TNTs (Translocants “NUP-TOP”). All of them were analyzed in details.

### Characterization of the Translocants

**Figure 3:**
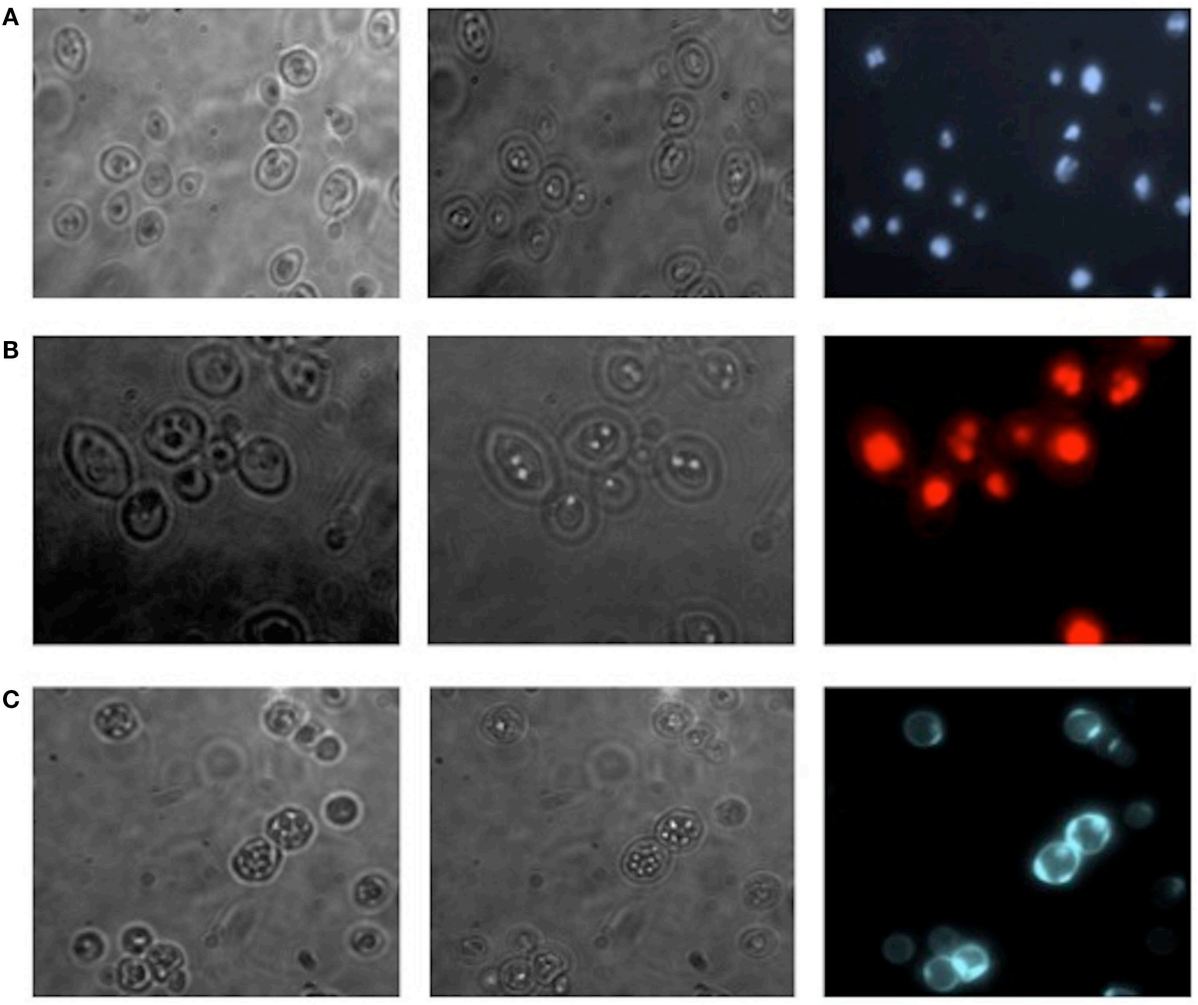
Fluorescent microscopy of aged (3 weeks old) NUP-TOP translocants. The spherical bodies, whose number increased with aging, can be visualized also without fluorescence using different focus lengths (the first two pictures of each panel), but they become more evident after staining with DAPI **(A)** and Pyronin Y **(B)**. After 4 weeks, all the cells of all the TNTs translocant strains contain a variable number of SBs. Cell aging is testified by the numerous scars on the cell wall that are visible after calcofluor treatment **(C)**.

**Figure 4:**
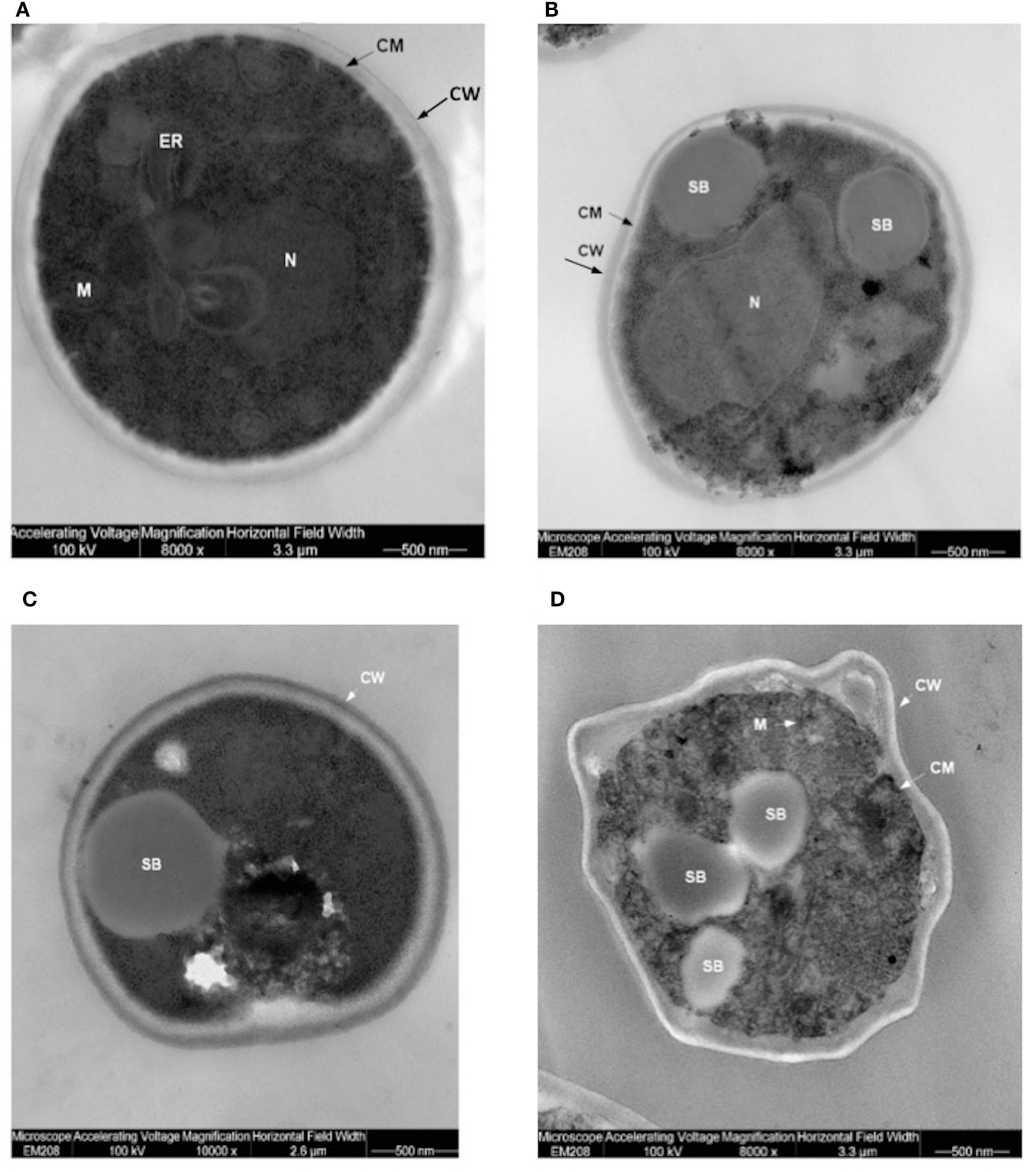
Trasmission electron microscopy (TEM) of aged (4 weeks old) translocants compared to the wild type (WT) strain: **(A)** WT San1, **(B)** TNT10, **(C)** TNT 10/P53, and **(D)** TNT 10/H273. N, nucleus; SB, spherical body; CW, cell wall; CM, cell membrane; M, mitochondria; ER, endoplasmic reticulum.

Translocants NUP-TOP were confirmed by DNA sequencing of the junction between chromosome VII and XIV and they were afterward analyzed by contour-clamped homogeneous electric field (CHEF) followed by Southern blot and sequential hybridizations (Figure S3 in Supplementary Material). Six out of nine translocants (clones 1, 2, 6, 7, 8, 10) showed a correct size of the translocated chromosome while three (clones 3, 4, 9) showed unexpected bands suggesting either spurious clones (as in clone 4) or abnormal rearrangements (for details, see the hybridizations panels of Figure S3 in Supplementary Material). The correct TNTs were further analyzed to check their phenotype while the expression of their chimeric transcript was verified by RT-PCR (Figure 2). The translocated chromosome was very stable also without selection, with 0.03% average of chromosome loss frequency in all the TNTs. Interestingly, when the translocants were left growing for at least 2 weeks, peculiar SBs started to mature within the cells. These structures have neither been found in other aged BIT yeast translocants (47) nor in human AML cells where only rod-shaped inclusions composed of fused lysosomes/primary neutrophilic granules, named Auer bodies, can be detected within the cytoplasm (48). DAPI (Figure 3A), and especially the RNA-specific dye PY (Figure 3B; Figure S4 in Supplementary Material), easily stained SBs, indicating an accumulation of RNA within the TNTs cells. The difference between the PY staining of the WT and of the translocants is remarkable (Figure S4 in Supplementary Material). To better investigate the SBs, a series of 4-week-old TNTs (Figure 3C) was observed in details by means of TEM. SBs appeared as big, interspersed cytoplasmic aggregates, lacking a surrounding membrane (Figure 4). The content of SBs is poorly electron dense, due probably to a progressive loss of material during the fixation procedure.

Spherical bodies are very different from stress granules as size, localization and number and are more similar to P-bodies (49) although they seem to condense into punctate patches (Figure 4).

### Genotypic and Phenotypic Investigation of the Translocation Loci

*NUP145* and *TOP2* are both essential genes in budding yeast and *NUP145* has been reported as haploinsufficient in rich medium (50). Synthetic lethality (BioGRID, Biological General Repository for Interaction Datasets) or genetic interactions (DRYGIN, Data Repository of Yeast Genetic Interactions) are not known between these two genes. Thus, to verify the phenotype of the NUP-TOP translocants in our genetic background, we performed the gene deletion of *NUP145* and *TOP2*. While *TOP2* deletion resulted, as expected, in a haploproficient phenotype (with approximately 90% of successful one-copy deletion), we confirmed that also in our genetic background *NUP145* is not only essential (51) but also haploinsufficient (50) since the heterozygous full gene deletion was never achieved even extending the homologies (data not shown). However, we succeeded in the partial deletion of one copy of *NUP145* with a frequency of 9.8%. Performing this deletion (with primers FwNupKlura, RevNUP-KO-Klura; Table S1 in Supplementary Material), a fragment of 861 bp at the 5′ end was left in homozygous condition, exactly as in the TNTs, suggesting that this gene portion is sufficient to avoid haploinsufficiency in rich medium. These data support early observations (52, 53) proposing that 200 bp at the 5′ end of *NUP145* are necessary to avoid haploinsufficiency in minimal medium (SC-LEU). We concluded that the 287 aa haplosufficient N-terminal region of Nup145, which contains also the GLFG (gly-leu-phe-gly) structural domain and which is part of the chimeric construct after the NUP-TOP translocation, is essential for diploid cells survival. Moreover, the overexpression of both *NUP145* and *TOP2* was verified in different background strains in the past (for a detailed description of the phenotypes and a list of references, see the Saccharomyces Genome Database at www.yeastgenome.org/), but the presence of the phenotype observed in TNTs was never reported.

To verify the possible influence of secondary structures on the fragility of the *NUP98* sequences, we performed extensive bioinformatics analyses of the region around the breakpoints to identify putative motifs responsible for non-B DNA conformations.

### Bioinformatic Analysis of the Breakpoints

The programme SCOPE—algorithm BEAM—(54) identified a direct, non-degenerate repeat (5′-ACTAGA-3′) leading to a slipped (hairpin) structure exactly at the breakpoint, within intron 13 (Chr. 11) of *NUP98* (Figure S5 in Supplementary Material). Running the same program with the algorithm SPACER it was possible to detect an inverted, degenerated repeat (5′-ACAAYRTTG-3′) within the breakpoint of intron 25 (Chr. 3) of *TOP2B* (data not shown). While the hairpin structure is responsible for non-B DNA, the degenerated inverted repeats are often found in rearrangement events in eukaryotes (55).

Since not only hairpins-prone repeats (56) but also repeated bending (57) can affect nicking at non-B DNA conformations and can, therefore, induce chromosomal translocations through non-homologous end joining (58), or homologous recombination (59) we analyzed the curvature and bending of the DNA within the *nup* locus.

**Figure 5:**
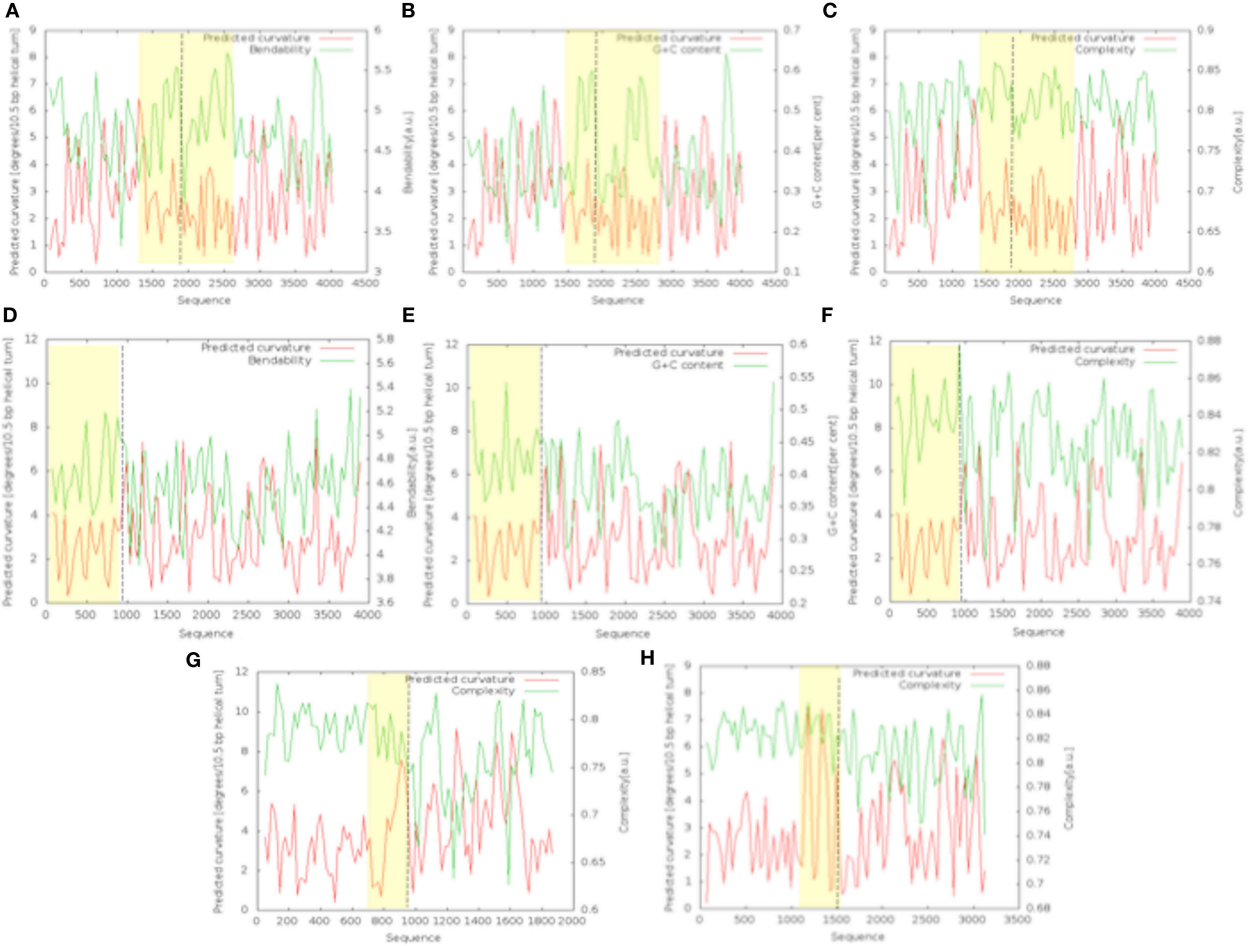
Bend.it analysis. The upper panel refers to the analysis of the genomic DNA around the breakpoint (in the figure at 1,982 bp) within *NUP98*. The panel in the middle refers to the analysis of the breakpoint (in the figure at 982 bp) within *NUP145*. **(A–C)** (*NUP98)* and **(D–F)** (*NUP145)* show the bendability, the GC content and the complexity of the genomic region against the curvature, respectively. The bottom panel refers to the comparison between the cDNA complexity of the junctions *NUP145*-*TOP2* **(G)** and *NUP98*-*TOP2B* **(H)**. The breakpoints in the graphics are at 883 bp **(G)** and 1,666 bp (H). In all the panels, the *Y*-axis is reported as degrees/10.5 bp helical turn. Dashed lines indicate the breakpoints while the peculiar regions discussed in the text are outlined in yellow.

**Figure 6:**
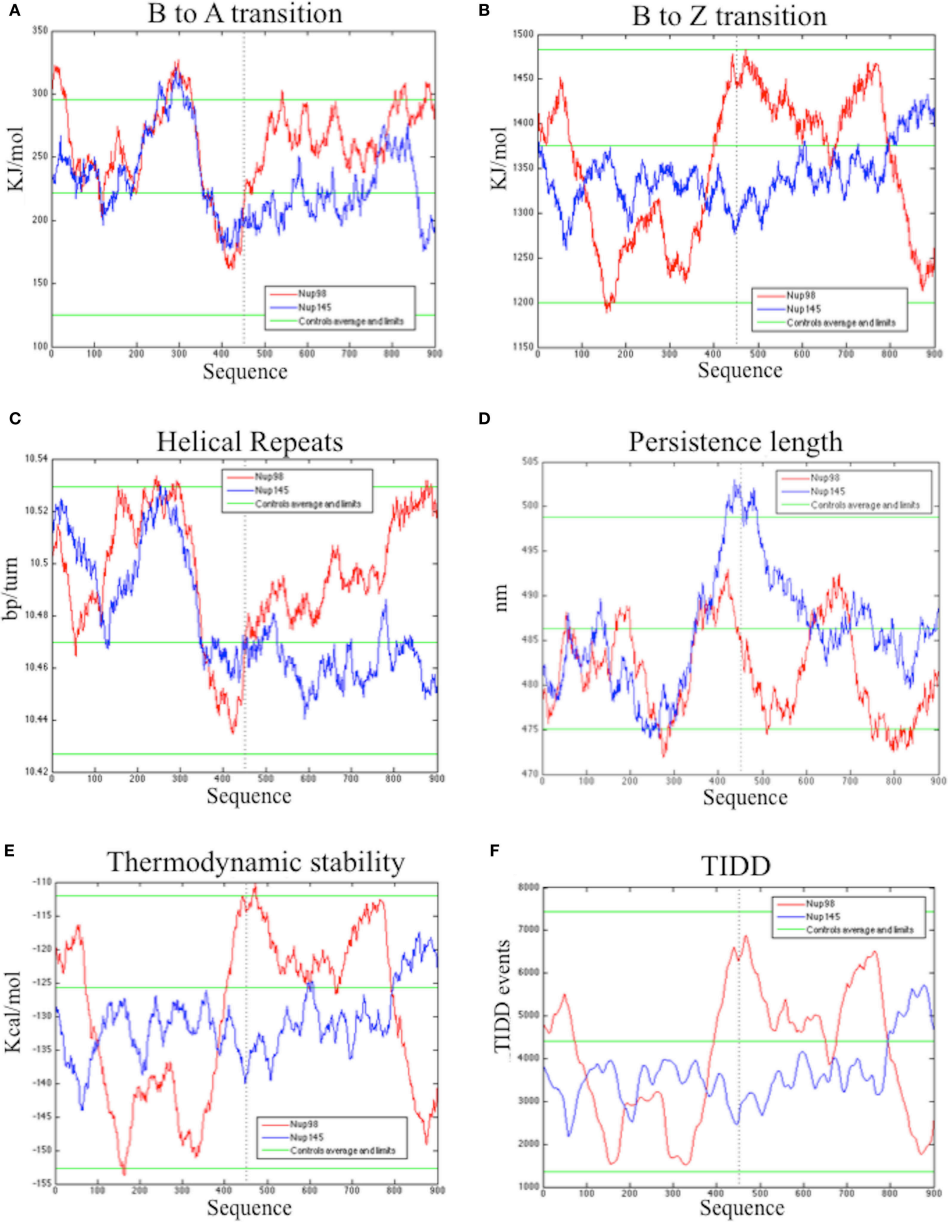
Bioinformatic analysis of the physicochemical and conformational properties of the sequences around the breakpoints of *NUP98* (red color) and *NUP145* loci (blue color). The three green lines in each graphic correspond to an average, maximum and minimum value of five control sequences (see Materials and Methods for details). **(A)** B to A transition, **(B)** B to Z transition, **(C)** Helical repeats, **(D)** Persistence length, **(E)** Thermodynamic stability, **(F)** thermally induced duplex destabilization (TIDD). The *X*-axis is labeled with numbers representing the nucleotide sequence; the *Y*-axis is labeled, in the different panels, with kiloJoule per mole (kJ/mol), nanometers (nm), kilocalories per mole (kcal/mol), base pairs per helical turn (bp/turn).

The bend.it analysis of *NUP98* genomic DNA revealed high GC content and high bending immediately before and after the region of the intron 13 that spontaneously breaks (Figures 5A–C). A sudden fall of bendability and a specular increase of curvature correspond exactly to the breakpoint. Similarly, *NUP145* shows high bendability, high GC content, and low curvature from the 5′ end until the breakpoint (Figures 5D–F). Then, after this, dramatic changes occur and the curvature suddenly increases. Therefore, the comparison of the DNA complexity in TNTs and in the translocant *NUP98-TOP2B* (Figures 5G,H) may predict the point of junction between the two chromosomes. Peaks of high GC content are usually associated with high thermostability and low homologous recombination (60) while fast re-associating DNA shows low complexity. High GC content, which correlates with high local bending, means also a denser, less flexible DNA and easy B-Z DNA transition. Analysis of computationally predicted physicochemical structural properties of *NUP98* showed that bulky changes occur around the breakpoint (Figure 6). In the profile of TIDD (37), the intensity of destabilizations increase of 80% around the breakpoint (in particular the number of TIDD events rise from 1,500 to 6,000, Figures 6F) and a similar trend could be observed in the predicted DNA thermodynamic stability with a 60% change (from −150 to −115 kcal/mol), in the B to Z transitions (from 1,270 to 1,450 kJ/mol) and in the B to A transitions (from 300 to −160 kJ/mol) with over 70% variations around the breakpoint (Figures 6E,A,B respectively). These results clearly and conjointly indicate an overall “weakening” of this genomic region. Conversely, *NUP145* did not display pronounced physicochemical changes around the homologous region of the artificially induced breakpoint except with predicted B to A transition (Figure 6A). However, conformational properties such as the helical repeats and the persistence length have a similar trend either within *NUP98* or *NUP145* (Figures 6C,D). In both genes, the persistence length increased from 475 to 490–500 nm while helical repeats decreased from approximately 10.52 bp/turn to 10.44–10.46 bp/turn immediately before the breakpoint (Figures 6C,D).

### Study of the Effect of Human *P53* Expression in TNTs

**Figure 7:**
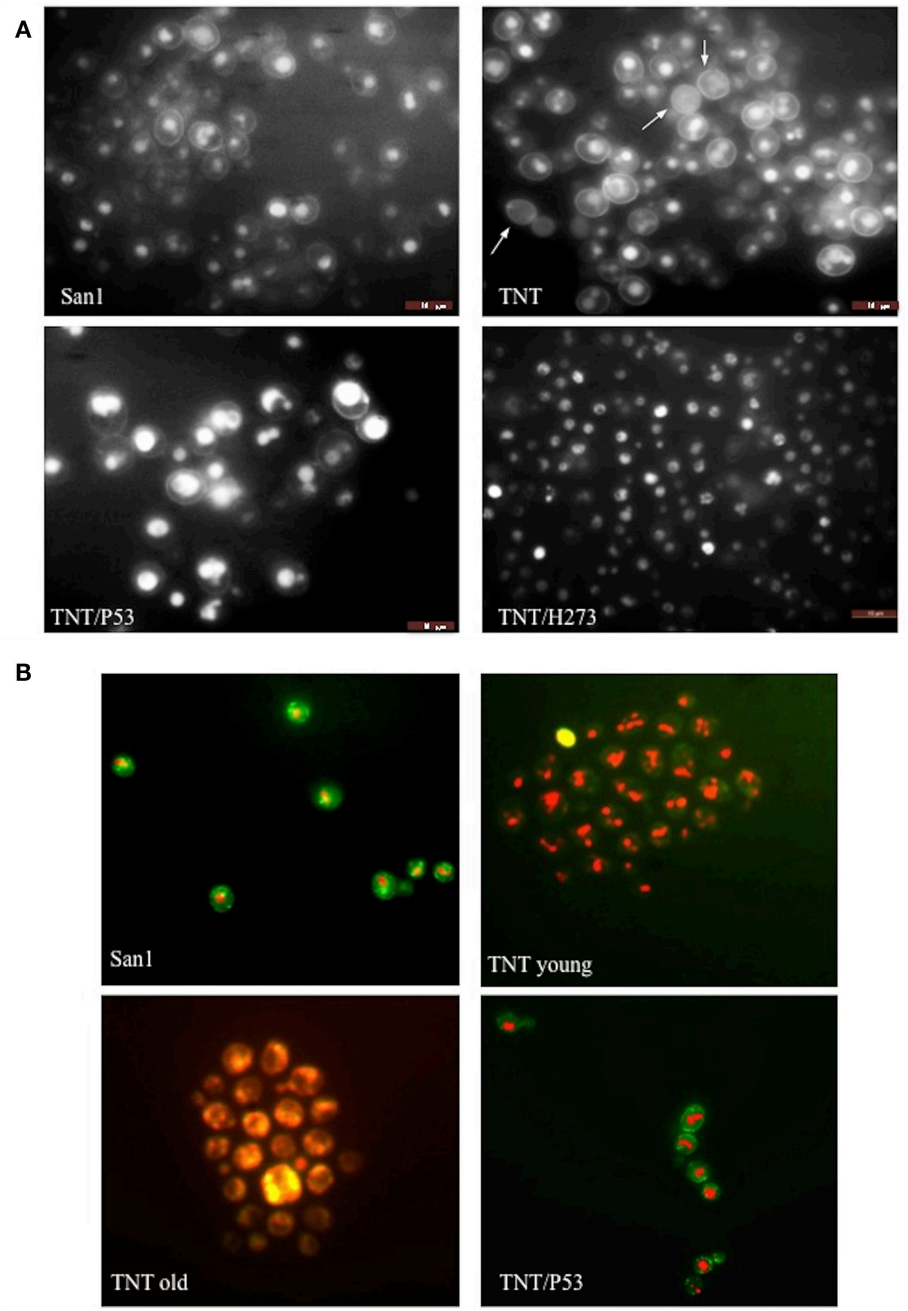
Fluorescent microscopy of **(A)** endocytosis **(B)** and cell viability. In **(A)**, lucifer yellow (LY) was used to test endocytosis in the wild type (WT) San1, in TNT cells, in TNTs transformed with P53 (TNT/P53) and with P53 mutated in H273 (TNT/H273). LY gives a high background staining of the cell wall of the translocants with a defective accumulation of the fluorescent molecule in the vacuole. The white arrows indicate cells with almost complete loss of endocytosis (roughly 10% of the cells). P53 restores a good level of endocytosis in the translocants. In **(B)**, the *FUN-1* staining reveals comparable viability of both San1 and the TNT cells transformed with P53. The TNT cells without P53 appeared already suffering when young cultures (2 days old) and very sick when old cultures (3 weeks old). The dead cells are stained in yellow. After 4 weeks, 5% of the cells appeared dead (yellow) and all them appeared as very sick (orange color).

**Figure 8:**
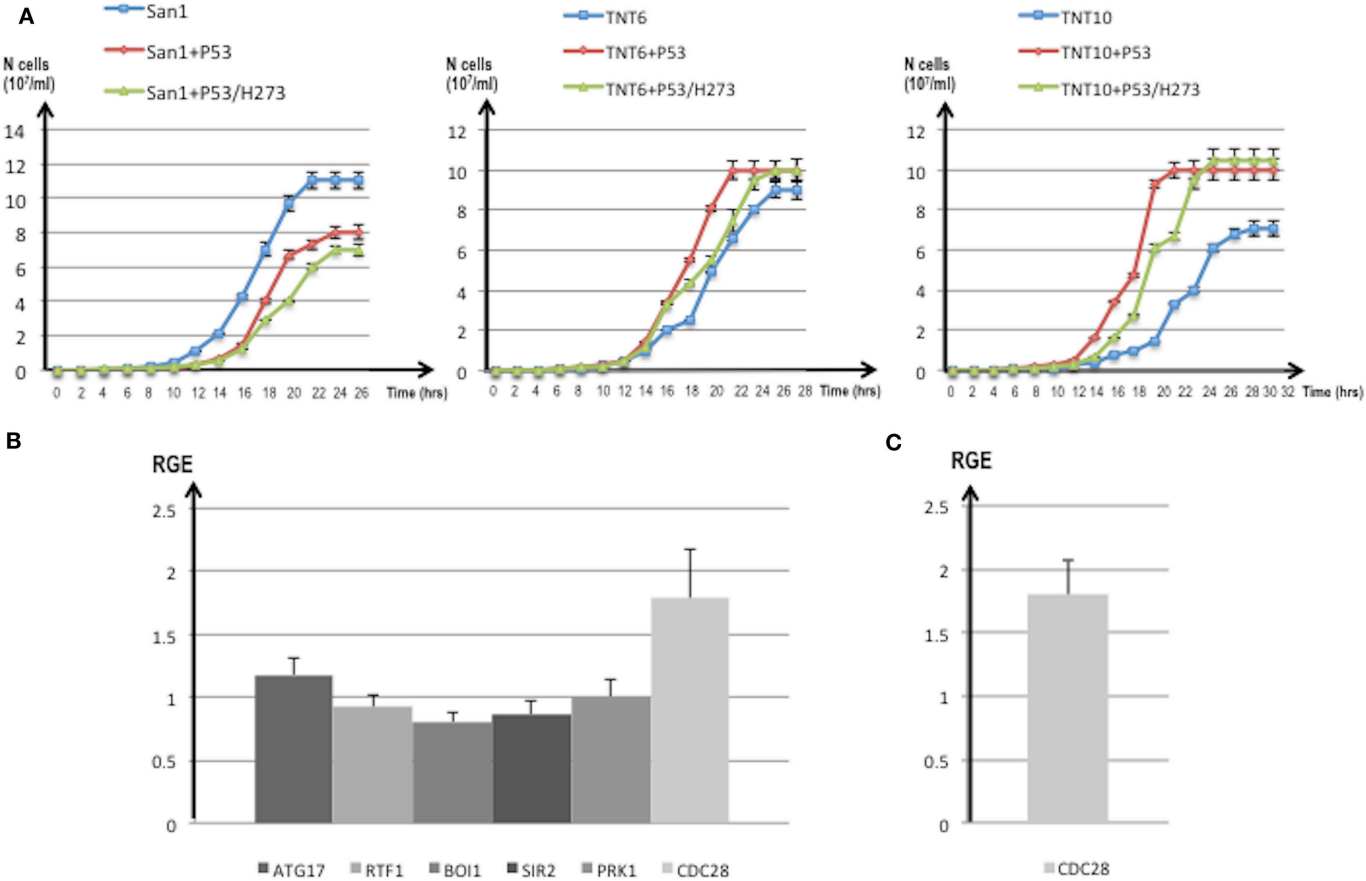
**(A)** Growth curves of translocants number 6 (TNT6) and 10 (TNT10) and of the wild type (WT) San1. The curves without P53 (blue), with P53 (red), and with the P53 mutant H273 (green) are shown for San1 (left), TNT6 (center), and TNT 10 (right panel). On the *X*-axis, the growth time (in hours), and on the *Y*-axis the cell number (10^7^/ml) are reported. Each aliquot was retrieved every 2 h and cell density determined by counting each aliquot three times on a Neubauer Improved Cell counting chamber. Error bars are reported accordingly. **(B)** Relative gene expression (RGE) of six genes (*ATG17, RTF1, BOI1, SIR2, PRK1, CDC28*) measured in TNT6 with P53. As reference, TNT6 transformed with an empty PJL49 plasmid was used (see Materials and Methods for details on the comparative method). *CDC28* increases 1.79-folds. **(C)** RGE of *CDC28* in San1 transformed with P53. The increase of *CDC28* is 1.8-folds respect to San1 transformed with an empty plasmid. The bars represent the average of three independent readings (bar errors are reported and calculated as standard error). **(D)** The expression of the mutant *P53/H273* was normalized respect to the WT form of *P53* taken as 1 in the WT strain San1 and in two different translocants (TNT6 and TNT10). RGE was calculated using *ACT1* as reference gene and following the procedure described in Section “Materials and Methods.” Error bars are reported accordingly.

In AML, the frequency of *P53* mutations ranges from 4 to 15% (61) and it is, therefore, very low if compared with other types of cancer, such as the high-grade serous carcinoma of the ovary, where *P53* mutation rates are close to 100%. Nevertheless, poor prognosis is usually associated with *P53* mutations in hematopoietic malignancies and in particular in myeloid leukemia (61). Recently, by using next-generation sequencing, frequent mutations of *P53, NOTCH1* and *ATM* have been identified in chronic lymphocytic leukemia (62). The P53 protein is usually mutated in the hotspot region of the DNA binding domain (aa 273–280) and in particular in the position H273. The high frequency (around 95%) of this mutation was also confirmed by a leukemic specific profile from a comprehensive analysis of 268 mutations of *P53* in 254 patients (63). Many abnormalities of the P53 network have been implicated in the pathogenesis of AML (64) and, moreover, the activity of nucleoporins (in particular Nup98) and both topoisomerases can be modulated by P53 (65–67). The observation that increased expression of P53 protein can be present in several types of human leukemia cells at different stages of differentiation, and in particular in AML (levels 10- to 100-fold those of fresh normal low-density human bone marrow cells), was reported a long time ago (21). More recently, other authors demonstrated that high levels of P53 protein carry an adverse prognosis, regardless of mutation status (22). More than half of patients with AML showed P53 protein expression by flow cytometry (68), P53 increased quantification in 256 AML patients was shown in proteomic profiling (69). Furthermore, it was assessed (24) that strong P53 expression in bone marrow progenitor cells was significantly associated with higher AML risk (*P* = 0.0006) and shorter survival (*P* = 0.00175) rendering P53 as the stronger predictor of transformation to AML (25). A *P53* ortholog seems not to be present, or at least has never been identified, in *S. cerevisiae*. However, the P53 pathway is very well conserved in yeast. Moreover, *S. cerevisiae* has proven to be an efficient model system for studies of the tumor suppressor P53 and in particular of its transcriptional activity (70), apoptosis induction (71) and modulation of the Warburg effect (18, 72) that are important prognosis predictors in leukemia (73). We, therefore, transformed the translocant yeast with a constitutive plasmid carrying human *P53*. The cDNA of P53 and of P53/H273 were cloned without the untranslated regions (UTRs), which may impair translation in yeast (23), in the constitutive vector pJL49 (see Materials and Methods). After the resultant constructs were sequenced TNTs were transformed with the pJL49 + P53 and pJL + P53/H273 constructs and also with an empty pJL49 vector to generate a negative control strain. We verified that P53 did not have any revertant effect on the TNTs phenotype since the SBs were clearly visible also when *P53* was expressed in the translocants (Figure 4C). Moreover, since it is known that P53 participates in the regulation of clathrin-mediated endocytosis (74) and since we demonstrated in the past that BIT translocants usually show impaired endocytosis (34), we tested endocytosis in TNTs with and without *P53* expression. P53 did not strongly modify the endocytosis in TNTs (Figure 7A), although it seemed to slightly improve it. These results correlate with an increased vitality of the P53-expressing translocants, as suggested by the fluorescent *FUN-1* assay on the cylindrical intra-vacuolar structures (Figure 7B). In effect, when TNT cells were transformed with P53 they showed a strong staining of the vacuole, similarly to the WT strain, indicating a vigorous and unexpected fitness (Figure 7B). To corroborate this hypothesis, we verified that the constitutive expression of *P53* favored the growth of the translocants albeit impairing the proliferation of the WT strain (Figure 8A). All BIT translocants usually grow less and at a lower density than the WT strains from which they derive (46), frequently showing endocytosis defects (34) and short chromosomal life span (47). It was already known that induced overexpression of human *P53* inhibits wild yeast proliferation probably because of its transcriptional activity on selective yeast genes involved in cell cycle arrest or cell death (23, 75, 76). Besides, it was demonstrated that Nup98 regulates the expression of P53 target genes in mammalian cells (77). Each *NUP98* fusion differs from the others with respect to P53 expression. For example, it is known that in case of *NUP98* fused with *HOXD13, JARID1A*, and *HOXA9*, the HOXA cluster genes are upregulated (5) and that *HOX5* under expression limits *P53* expression in tumors (78). Moreover, the complete loss of one or both alleles of *P53* can accelerate the development of AML in a *NUP98–HOXD13* mouse model (79). On the contrary, topoisomerase II interacts directly with the C-terminal region of P53 (80), although we presently ignore how this interaction can be affected in the *NUP98-TOP2* translocation.

For these reasons, we compared the expression of putative P53 targets in TNTs translocants with and without P53 expression. We decided to quantify the expression of six yeast genes that code for proteins directly interacting with P53 like the autophagy-related Atg17 (81) and the chromatin remodeling Rtf1 (82) or that are orthologs of human regulators of P53 such as Sir2 (83), Pak1/Prk1 (84), Mtbp/Boi1 (85), and the cyclin-dependent kinase Cdc28 (86). The majority of these genes were similarly expressed in the translocants with and without P53 expression (Figure 8B); nevertheless, a substantial increase (1.79-folds) of the *CDC28* transcript was detectable in P53-expressing translocants (Figure 8B). To verify whether this increase was a peculiarity of the translocants, we measured the *CDC28* expression in the WT strain transformed with *P53*. Also in this case, an increase of 1.8-folds of *CDC28* expression was detected (Figure 8C) suggesting that the effect of P53 on *CDC28* is independent on the *NUP-TOP* translocation effect. The expression of *P53* and of the mutant *P53/H273* was verified in two different TNT strains. The two genes were expressed at comparable level in both TNTs and in the WT strain (Figure 8D).

## Discussion

The nucleoporin Nup98 is an essential component of the nuclear pore complex (NPC) and takes part into the nuclear-cytoplasmic traffic, including mRNA export. Several chromosomal rearrangements such as translocations and inversions, with 28 consequent Nup98 gene fusions, are associated with a wide array of hematopoietic malignancies (5). Because in the past we developed the BIT system to generate *ad hoc* translocations without strain pre-engineering, just by exploiting the strong homologous recombination of yeast cells (11, 87), we decided to reproduce in *S. cerevisiae* a deeply characterized translocation in humans, responsible of AML, between *NUP98* and *TOP2B* (13). This modeling of the translocation event allowed us to investigate the genetic etiology of AML that can be affected by the physico-chemical properties of the genomic regions around the breakpoints and by the so-called position effect (88) or position-effect variegation (89). Before triggering the translocation between the yeast orthologs *NUP145* and *TOP2*, we investigated the DNA conformational properties around the *NUP145* breakpoint, comparing them with those of *NUP98* (Figure 5; Figure S5 in Supplementary Material). It is presently not known why the genomic sequences of *NUP98* are so prone to break, but it has been postulated that introns act as recombination enhancers within coding sequences, increasing the efficiency of selection at nearby sites. Hotspots can be the results of an antagonistic co-evolution between distinct, but tightly linked, DSB inducers and DSB-cut regions (90). It is well known that many of the translocation breakpoints are within a region of predicted non-B DNA conformation. Cruciforms, triplexes, hairpins, slipped conformations, quadruplexes, and left-handed Z-DNA are formed by repeats in these regions and are usually responsible for genomic instability leading to translocations, inversions, deletions, or insertions [for an extensive review, see Ref. (91)]. The bendability is a local parameter representing the ability of DNA to bend (usually toward the major groove) as a result of thermal fluctuations (41) or DNA–protein interactions such as the one with P53 (92). Bended segments are usually associated with active transcription and play a role in chromatin organization by influencing nucleosome positioning (93). Comparable features of high bending and very low curvature before a breakpoint can be detected in both *NUP145* and *NUP98*. These results are in agreement with preliminary observations indicating that high bending is a sign of genome integrity (94). In our analysis, a strong bending drop as well as an 80% increase of TIDD events between bent segments can both predict the exact breakpoint (Figures 5 and 6). These data agree with the typical straight conformation of other fragile sites that are characterized by poor thermal stability and are flanked either side by highly bent DNA segments (95). Bending strongly affects recombination, especially site-specific recombination (96, 97), and, resulting as Z-DNA formation (98), it can thus affect BIT efficiency. BIT has usually a frequency varying from 4 to 10% using homologies of 65 nt (11). Its frequency variability depends primarily on the genomic sites chosen as targets. It is much easier to target inter-genic regions, promoters, terminators, pre-telomeric sequences (17, 19) and *vice versa* it is difficult to target intragenic or GC rich regions (Tosato and Noel, personal communication). Usually, coding regions correlate not only with low recombination rates but also with weak nucleosome positioning and strong DNA complexity patterns (99) that were in fact detected at the 5′end of *NUP145* (Figures 5G). The *nup-top* translocation represents in this sense an exception because using a standard BIT cassette, with 65 nt of homology and no repeats, the translocation frequency was high (8.3%; Table S2 in Supplementary Material) despite the targeting of intragenic regions with high GC content (Figure 5). In particular, the locus *top2* seemed to be the hotspot for recombination (Table S2 in Supplementary Material). The initial difficulty to find a stable TNT (0.6%) was, therefore, due to the artificial repeated region added to the cassette for the POP-OUT induction and not to the poor recombinogenicity of the genomic targets, as also testified by the strong intra-recombination of 5′end *NUP145* segments leading to the concatemer. We, thus, demonstrated in this work that it is feasible, although with low frequency, to obtain a perfect fusion *in vivo*, without any DNA sequence scar, between two selected genomic loci exploiting BIT followed by a selectable POP-OUT of the marker.

When *P53* was expressed in the NUP-TOP translocant yeast, BIT was still possible even if with lower frequency, but there was a different distribution of integration events. With a constitutive expression of *P53*, ectopic integrations (obtained regardless of the homology) more than doubled (Table S2 in Supplementary Material). It is well documented that P53 suppresses homologous recombination and modulates the recombination pathways (100, 101), giving an explanation to the high rate of ectopic integrants that were found when P53 is expressed (Table S2 in Supplementary Material). P53 seems, moreover, to be less toxic for BIT translocants than for the WT (Figure 7) and its presence is almost beneficial for the translocants, increasing their vitality (Figure 8A). The toxicity of P53 in yeast is mainly related to gene repression of thioredoxin (Trx1/2), a highly conserved multifunctional anti-oxidative and anti-apoptotic protein family required for the detoxification of reactive oxygen species (ROS) (23). *P53* protects against metabolic stress by upregulation of oxidative phosphorylation and modulation of antioxidants (102) and, when expressed in yeast, induces ROS accumulation, which represents the major cause of cell death (23). We previously demonstrated that BIT translocants have a very high deregulated oxidative stress response network resulting in extremely high and persistent ROS levels (47) and that BIT may induce adaptation with improved phenotypic fitness with respect to stressful conditions (46). We, therefore, speculate that all the ROS-related toxic effects of P53 could be negligible in adapted translocant cells. This theory is supported by our findings on the *CDC28* gene that is overexpressed in TNTs/P53. The increase in the levels of *CDC28* expression is present in cells that are able to re-enter the cell cycle more efficiently after stress (103). In order to verify whether the stress in our cells was given by the expression of *P53* or by the translocation event *per se*, we measured the level of expression of *CDC28* in the WT overexpressing *P53*. The overexpression was the same in the WT and in the translocants suggesting that the constitutive expression of *P53* is generally stressful for the yeast cell, independently of the translocation event and that P53 does not further worsen the stressful condition of the translocants. On the other hand, we noticed that the expression of human *P53* did not considerably affect the vitality (and the transformability) of the WT yeast strain subjected to BIT transformation (Table S2 in Supplementary Material). We know from previous studies that heat shock, required for DNA uptake, induces a transient G1 arrest in yeast cells for a period of approximately 1 h (104), and that once the heat shock proteins are induced and thermo-tolerance is acquired, the normal cell cycle resumes (105). Normal cell cycle progression in yeast relies on activation of the cyclin-dependent kinase Cdc28 and plasmid-driven overexpression of *CDC28* can suppress delay on cell cycle progression observed upon stress (103). Therefore, we can postulate that the long-lasting induction of *CDC28* by P53 is beneficial for stressed cells to recover after heat shock to re-enter the cell cycle more efficiently after DNA transformation.

Finally, neither P53 nor its H273-mutant was able to rescue the unusual phenotype typical of the aged translocants. SBs started to appear in TNTs as interspersed cytoplasmic aggregates, without any membrane, after 3 weeks of continuous growth. In budding yeast, as in higher eukaryotes, processing bodies (P-bodies) are dynamic *foci* within the cytoplasm that are not solid aggregates, as the stress granules, but liquid-like droplets (106) containing untranslating mRNAs and proteins involved in mRNA decay (107). Their size depends on the extension of defects in mRNA decapping and, more generally, to environmental perturbations (108). However, in the case of cellular stress, the size usually varies from 0.1 to 0.3 µm^2^ (106) that is comparable with the size of the SBs observed within the aged TNTs (Figure 4). Recently, it has been demonstrated that the *C. elegans* germ P-granules, which share a lot of similarities with P-bodies, associate with Nup98 and need an intact Nup98 for integrity and function (109). We still do not know whether the Nup98 ortholog, Nup145, is as well important for P-bodies in yeast, but our study indicates a role of this nucleoporin outside the nucleus and related to RNA-rich bodies within the cytoplasm. Nup98 is already known to be a tumor suppressor because it stabilizes P53 target genes (77). Here, we propose that its oncogenic properties could also involve dysregulation of RNA turnover in the cytoplasm supporting the hypothesis that P-body disassembly and subsequent mRNA deregulation might be linked to certain types of cancers (110). However, notwithstanding many studies, the exact mechanism by which P-bodies impact the development and progression of cancer is largely unknown and a thorough understanding of their roles in carcinogenesis could help in the identification of new targets for cancer therapy (111).

## Conclusion

Our data suggest that Nup98 could be related to P-bodies regulation in yeast and, therefore, be responsible for mRNA turnover in the cytoplasm. We suppose that other leukemic translocations involving Nup98 might be characterized by the same defects of cytoplasmic mRNA dysregulation. We confirmed that, like *NUP98* in humans, *NUP145* is haploinsufficient in yeast. It is likely that this *NUP-TOP* induced translocation generates secondary chromosomal rearrangements as we demonstrated in the past for other BIT events (17) and as shown by the Southern hybridizations of the translocated clones (Figure S3 in Supplementary Material). Possibly, secondary aneuploidies resulting from *NUP145* haploinsufficiency could generate genome instability, as already surmised for *NUP98* translocations in human cells (5). Last, but certainly not least, this work points out a role of *P53* in these Nup98-translocated cells, although the inactivation of *P53* is a frequent event in tumorigenesis (61, 71). Here, we demonstrated that in the yeast model expression of *P53* improved vitality, endocytosis and growth of translocated cells, fostering considerations on its possible role in translocation-related tumors.

## Author Contributions

VT and DN had the idea and conceived the strategies; VT designed and performed the majority of the experiments; NW helped in the PCRs and microscopy; JZ performed the bioinformatics analysis; GS and RM contributed to the P53 data; VT, MB, and CB analyzed the data and wrote the paper.

## Conflict of Interest Statement

The authors declare that the research was conducted in the absence of any commercial or financial relationship that could be construed as a potential conflict of interest.

## Footnotes

^1^http://www.focusonmicroscopy.org/2011/PDF/412_Rybak.pdf.

^2^http://tidd.immt.eu.

## Abbreviations

BIT, bridge-induced translocation; AML, acute myeloid leukemia; HRS, homologous recombination system; 5-FOA, 5-fluoroorotic acid; TEM, transmission electron microscopy; TNT, translocant NUP-TOP; NPC, nuclear pore complex; TIDD, thermally induced duplex destabilization; SB, spherical body.
