# Supplementary material for: Bridge-Induced Translocation between NUP145 and TOP2 Yeast Genes Models the Genetic Fusion between the Human Orthologs Associated With Acute Myeloid Leukemia
Source: Front Oncol. 2017 Sep 29;7:231. doi: 10.3389/fonc.2017.00231 (PMC5626878; doi:10.3389/fonc.2017.00231)
Supplement: Supplementary file 1 [file table_1.docx]

**TableS1. Primers used in this work**

Primers utilized in BIT to amplify the cassette and to verify the integration (the asterisk indicates the primers used also in the RT-PCR and in the amplification of the scar):

FwNUPKlura: CGTTTTCTTCCTCAGTTTCAGGGAACGCTCCTTTACCACTCGCCTCACAGTCAAGTTTAGTGAGTcgtacgctgcaggtcgacgg (Homology on Chr. VII: 338723-338787)

RevTOP-NUPKlura:

ACCATTTATAACATTTTCAGTATCTTCGTGTGAACTTTCTTCATCTTCTTCATCAGAAGTACTCACTAAACTTGACTGTGAGGCGAGTGGTAAAGGAGCGcccgcgcgttggccgattca (Homology on Chr. VII: 338748-338787; Homology on Chr. XIV: 460959-461018)

TOPREV-Klura:

CTTCAGGACCATTTATAACATTTTCAGTATCTTCGTGTGAACTTTCTTCATCTTCTTCATCAGAAGTcccgcgcgttggccgattca (Homology on ChrXIV: 460959-461025)

FwNUP*: TGTTCGGTAAGCCTACTACAA (Chr. VII: 338369-338389)

RevNUP: CCATTCTGAGGAAAGATGCTG (Chr. VII: 338946-338966)

NupFwUP: ATGCCACAGCAAATTCTACGG (Chr. VII: 338234-338254)

NupFwDown: GTAATGTTCCTATGGCAGTGG (Chr. VII: 338609-338629)

FwTOP: GACCATATGAGCGAAAGGTTAC (Chr. XIV: 460731-460752)

RevTOP*: TATCCCACCTCAAAAGCCTTC (Chr. XIV: 461180-461200)

RevTOPII: GGTCAATGACCATATTCTCATTTC (Chr. XIV: 461055-461078)

Klura1: ACCGGACTTGCATGAGTCTC

Klura2: TTTGATTGGCTAATCATGACCC

NUPSacI: ACCGAGCTCCCAGTTCCCTTCAATTCCCT (VII: 337988-338007)

NUPEcoRI: GGAATTCCCTGACTAAACTTGACTGTG (VII: 338768-338785)

FwNUP100: GGAACCCAAACCAATTGAAAATAGAAGAACATATTCGTTTTCTTCCTCAGTTTCAGGGAACGCTCCTTTACCACTCGCCTCACAGTCAAGTTTAGTGAGTcgtacgctgcaggtcgacgg (VII: 338687-338787)

RevTOP_NUP800:

TGTGCCATATAGTTCTTCAGGACCATTTATAACATTTTCAGTATCTTCGTGTGAACTTTCTTCATCTTCTTCATCAGAAGT*ACTCACTAAACTTGACTGTGAGGC*

(XIV: 460959-461039, *in italic the homology on NUP145*)

FwNUP-KO-Klura:

RevNUP-KO-Klura:

TTATATCTTATATGTACACTTCATTAAATCTGGGCAAACTCcccgcgcgttggccgattca

F1-KO-NUP: ATGATCAGTATAAGCACTGTC (Chr.VII: 341654-341674)

R1-KO-NUP: TTAGACCTACTGAAGAACACT (Chr.VII: 341955- 341975)

FwPstNUP300: AACTGCAGCGACAAACTCAACGACAGG (ChrVII: 338429-338447)

RevBamNUP300: AAGGATCCAAACTCACTAAACTTGACTGTG (Chr. VII: 338768-338787)

FwSacNUP150: ACCGAGCTCGACATGCCAAGATCTATTAC (Chr. VII: 338633-338651)

RevEcoNUP150: GGAATTCCACTCACTAAACTTGACTGTG (Chr. VII: 338768-338787)

Primers used to amplify probes:

KluraDig1: CTGAGACTCATGCAAGTCCG (within the Kl-URA3 gene)

KluraDig2: ACCTGGGGTCATGATTAGCC (within the Kl-URA3 gene)

KluraPromDig1: GAGTGTATTGACGCTGGCGT (within the Kl-URA3 promoter)

KluraPromDig2: GTGATTCTGGGTAGAAGATCG (within the Kl-URA3 promoter)

BUD17Fw: GTGCTCTCCATTCAATCACAC (Chr. XIV: 674953-674973)

BUD17Rev: CGTACAGCCTTCCATTATCG (Chr. XIV: 675288-675307)

PAN2Fw: CTCACTAGCATTGATATTGC (Chr. VII: 334131-334150)

PAN2Rev: CCCATGTTGTTTGATGATCT (Chr. VII: 334471-334490)

RAD54Fw: AGAGATATTTACCAATTTGGCCC (Chr. VII: 193554-576)

RAD54Rev: CTTAAGTTCTAAAGCAGGTGGG (Chr. VII: 194196-194217)

GLR1Fw: TCTGATGGGTTCTTTAGATTGGAAG (Chr. XVI: 376060-376084)

GLR1Rev: AGTAACCAATTCTTCTGCGCTAGTC (Chr. XVI: 376920-376944)

Primers used to label the translocated chromosome with Kanamycin:

FwXIVKAN: CCATCAGGATTATCGTTCTCTGACATCACCCACTCTCTGCgtcgacggatccccgggttaa (homology on Chr. XIV: 652023-652062 - *KAN* homology in lower case)

RevXIVKAN: AAATCTGAAACTTGCAGATCATTAGTTTACGCTTCGGATGccgcgcgttggccgattcat

(homology on Chr. XIV: 652394-652433- *KAN* homology in lower case)

F1XIV: CATACATGCAACACATACTC (Chr. XIV: 651836-651855)

R1XIV: GTTCTTTTGCTTTTGATCCG (Chr. XIV: 652199-652218)

F2XIV: AAAGCCATCACGTGGATCCC (Chr. XIV: 651900-651919)

R2XIV: AGCAGGCTTTAGATGTAGCAG (Chr. XIV: 652589-652609)

Primers used for copy number determination:

Klura_CN: GCAAATGTATGGACCCAACGT (used with KluraDig1)

*P53*-cloning primers:

FwP53Bam: CGGGATCCCGATGGAGGAGCCGCAGTCAGA

RevP53Eco: GGAATTCCTCAGTCTGAGTCAGGCCCTTC
