# Supplementary material for: Bridge-Induced Translocation between NUP145 and TOP2 Yeast Genes Models the Genetic Fusion between the Human Orthologs Associated With Acute Myeloid Leukemia
Source: Front Oncol. 2017 Sep 29;7:231. doi: 10.3389/fonc.2017.00231 (PMC5626878; doi:10.3389/fonc.2017.00231)
Supplement: Supplementary file 2 [file table_2.docx]

**TableS2.** **Distribution of integration events after BIT transformation**

|  | Integrations in *nup* % | Integrations in *top* % | Ectopics % | Translocants % |
| --- | --- | --- | --- | --- |
| 1 | -- | 47.2 | 44.5 | 8.3 |
| 2 | -- | -- | 97.3 | 2.7 |
| 3 | 6.8 | 22.9 | 70.3 | -- |
| 4 | 15.8 | 16.9 | 66.7 | 0.57 |

The distribution of integration events in percentages (from left to right: integration in *nup locus* only, in *top locus* only, in ectopic locations, in both *loci* = translocation). The different rows refer to a wild type strain transformed with: 1: a standard BIT cassette with 65 bp of homology; 2: a standard BIT cassette with 65 bp of homology when *P53* is expressed; 3: a BIT cassette with 65 bp of homology plus 40 nt of repeated region of *NUP145*; 4: a BIT cassette with 100 bp of homology to target *nup* and 80bp to target *top* and a repeated region of 150 bp. Approximately four hundred colonies were analyzed to calculate the frequencies reported above.
