# Supplementary material for: Bridge-Induced Translocation between NUP145 and TOP2 Yeast Genes Models the Genetic Fusion between the Human Orthologs Associated With Acute Myeloid Leukemia
Source: Front Oncol. 2017 Sep 29;7:231. doi: 10.3389/fonc.2017.00231 (PMC5626878; doi:10.3389/fonc.2017.00231)
Supplement: Supplementary file 3 [file data_sheet_1.docx]

**Supplementary Figures Legends**

**Figure S1**

1. The scheme of the translocation *nup-top* is reported. The primers used to amplify the bridge (FwNUP, RevTOP) are drawn in violet. Their sequence is described in Table S1. For illustration purposes, the size of chromosomes and of genes is out of scale.
2. A scheme of the concatamer is reported. For illustration purposes, only three copies of the cassette are drawn. A green circle outlines the two primers k1 and k2 that were used to confirm the concatamer formation.
3. CHEF (left) and Southern hybridization (right) of several clones coming from the same BIT event. The probe used for the detection targeted the marker KlURA and was amplified using the primers KluraDig1 and KluraDig2 (TableS1). The arrow indicates the correct size of the translocant (664,579 bp) in the sample 112.
4. Templates with different stretches of homologies cloned in the plasmid pFA6aKlura to generate different BIT cassettes. 320 bp of *NUP145* were cloned PstI/BamHI upstream the *KlURA* gene while pieces from 150 to 800 bp were cloned SacI/EcoRI downstream the same gene. Several combinations of primers (sequences in Table S1) were used to amplify different cassettes. As example, a couple of these combinations are reported in the figure (above and below the template). The homologies (tails and primers) toward *NUP145* and *TOP2* are red and blue, respectively.

**Figure S2**

Targeting of the translocated chromosome with kanamycin.

1. Pulsed field gel electrophoresis (left) and its relative hybridization (right) with a probe against kanamycin. 1 and 2 are the control (wild type strains) while slots from 3 to 11 are different clones picked up from a targeting experiment. Samples 3 and 9 had the insertion of the cassette within the translocated chromosome (T).
2. The region of chromosome XIV that was targeted with the cassette carrying kanamycin is outlined. A yellow triangle indicates the exact insertion point of the kanamycin marker between the genes *PHO91* and *YNR014W*. The green lightning bolt marks the BIT translocation breakpoint within *TOP2.*

**Figure S3**

Sequential hybridizations of nine different translocants with probes against kanamycin (*KAN*), *RAD54, BUD17* and *PAN2,* respectively. The exact position of these genes is shown in the scheme on the top of the figure. The chromosomes size is indicated on the right of the panels to better visualize the hybridization bands. 1: clone 3; 2: clone 6; 3: clone 9; 4: clone 10; 5: wild type strain San1; 6: clone1; 7: clone 2; 8: clone 4; 9: clone 7; 10: clone 8. An arrow indicates the translocant.

**Figure S4**

Pyronin Y staining of the wild type strain San1 (A) and of a translocant (B). While in the wild type cells the red color fades and diffuses (A), in the translocants it localizes within the spherical bodies (B). A detail of the B image (white square) is shown in C. In all the panels the gray images (left) were taken without fluorescence. Details on staining protocols and used wavelengths are specified in M&M.

**Figure S5**

Bend.it analysis of the genomic sequence around the breakpoint of *NUP98*. In the figure, the bendability (above) and the G+C content (below) are reported against the curvature. The black arrow indicates the breakpoint. The programme SCOPE – algorithm BEAM - identified a direct, non-degenerate repeat (5’-ACTAGA-3’) leading to a slipped (hairpin) structure exactly at the breakpoint.
