# Supplementary figures and images for: Bridge-Induced Translocation between NUP145 and TOP2 Yeast Genes Models the Genetic Fusion between the Human Orthologs Associated With Acute Myeloid Leukemia
Source: Front Oncol. 2017 Sep 29;7:231. doi: 10.3389/fonc.2017.00231 (PMC5626878; doi:10.3389/fonc.2017.00231)

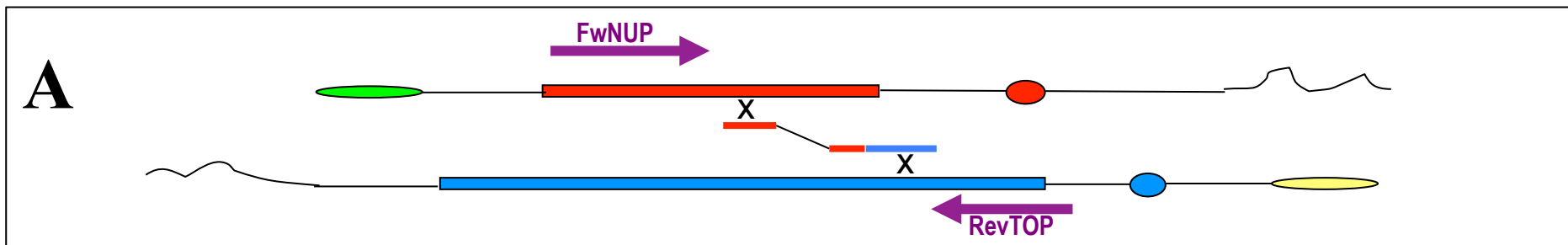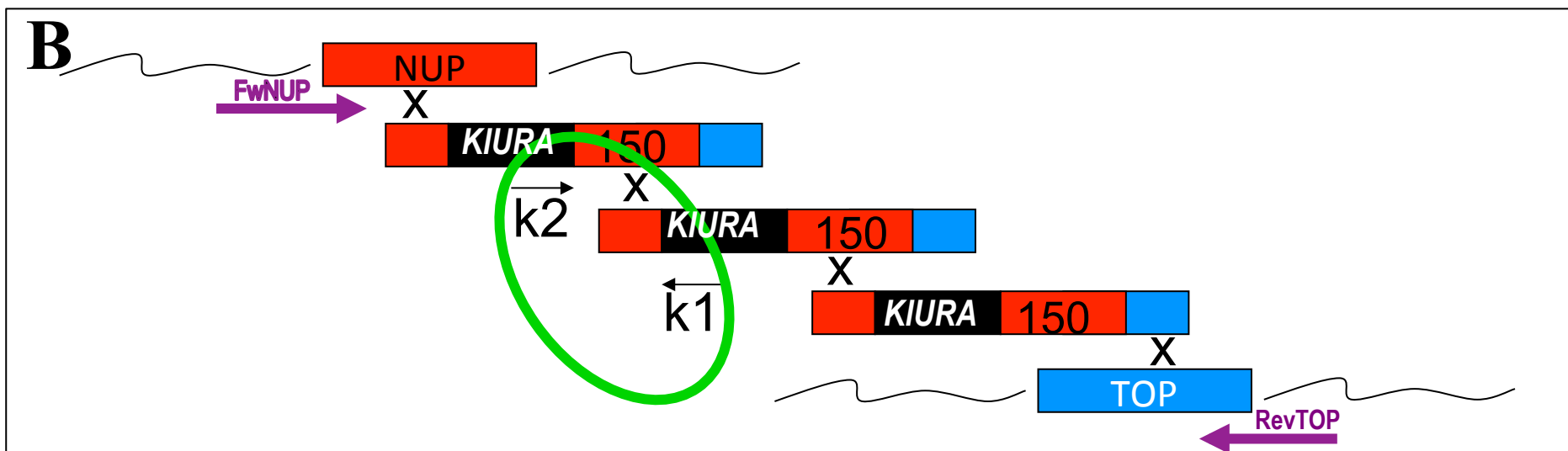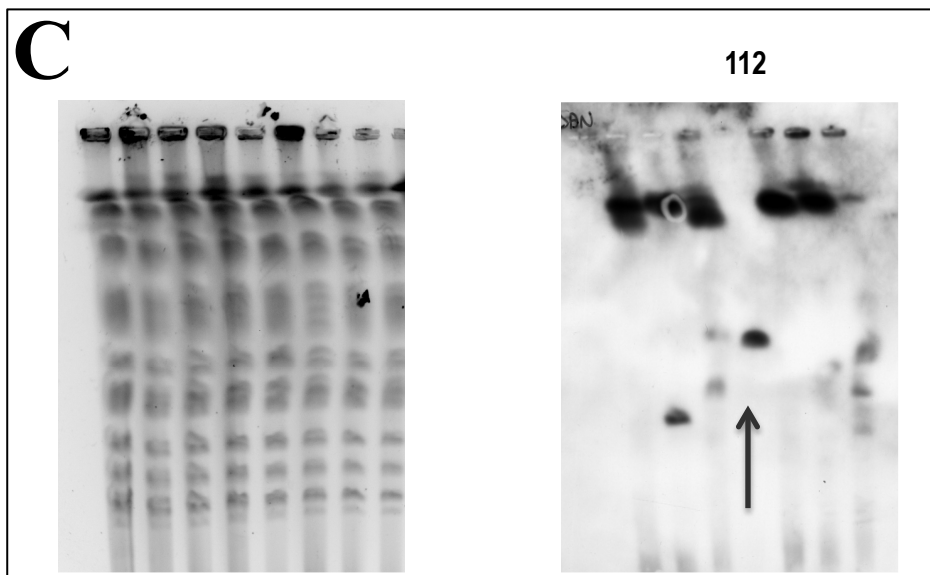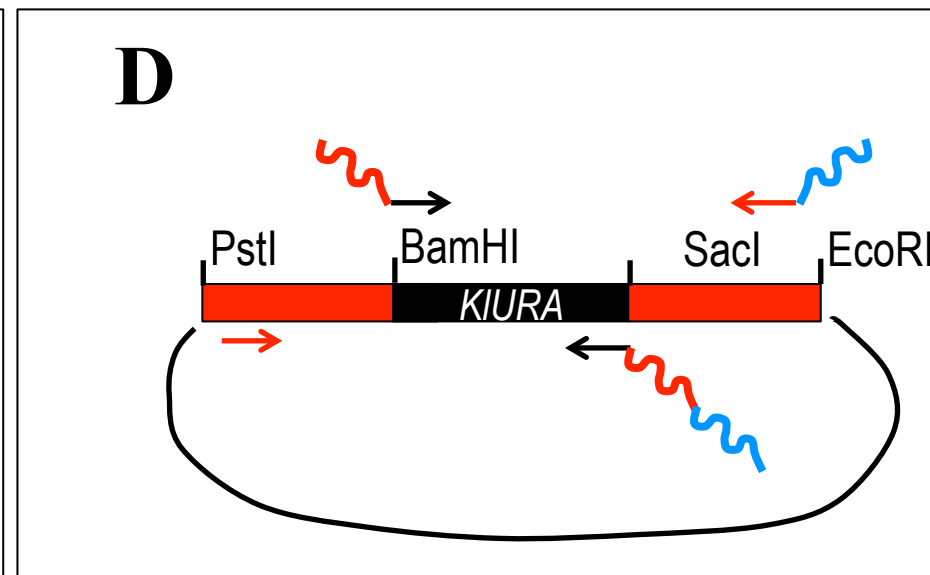

Supplement: Supplementary file 4 [file image_1.pdf]

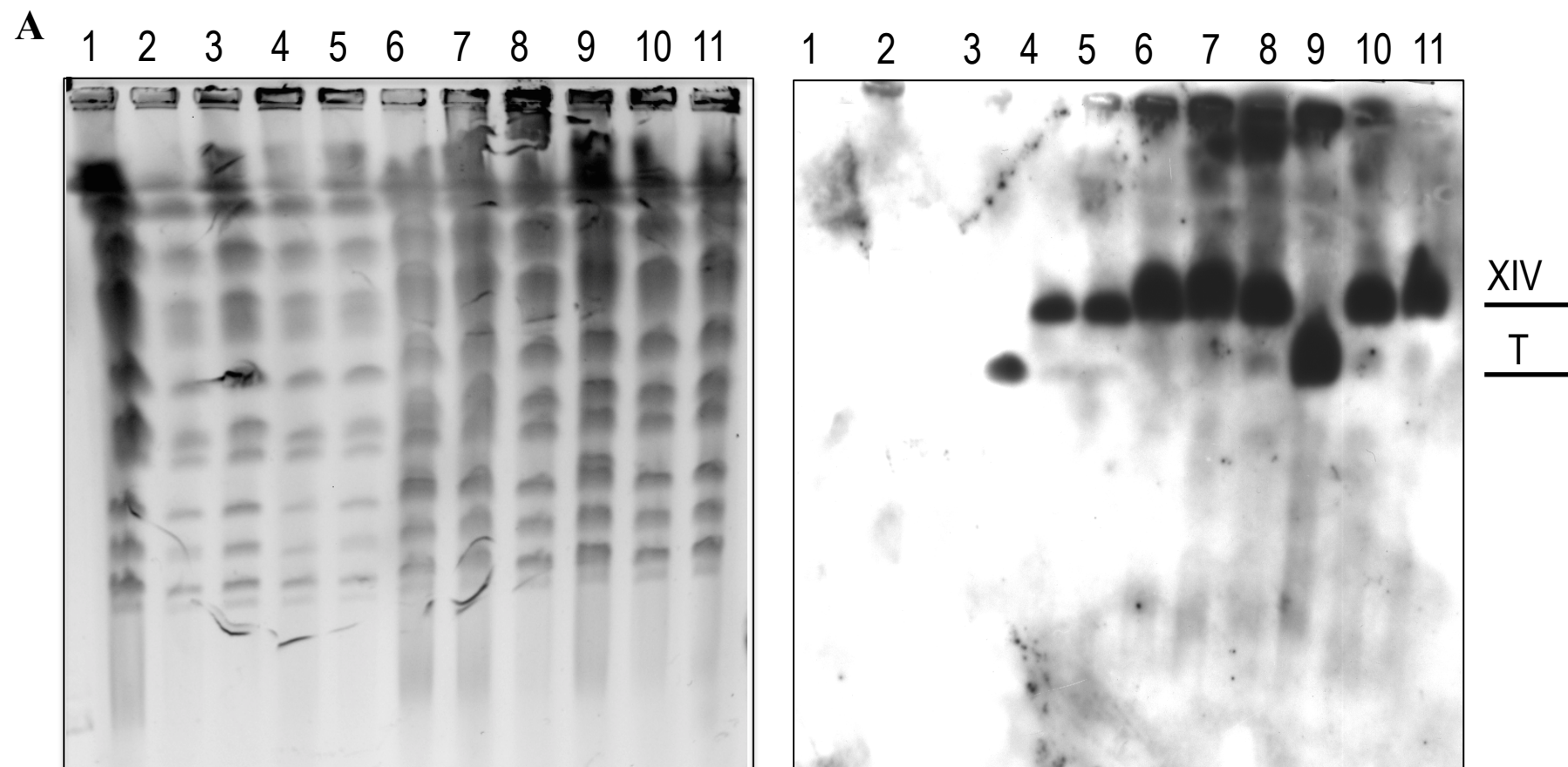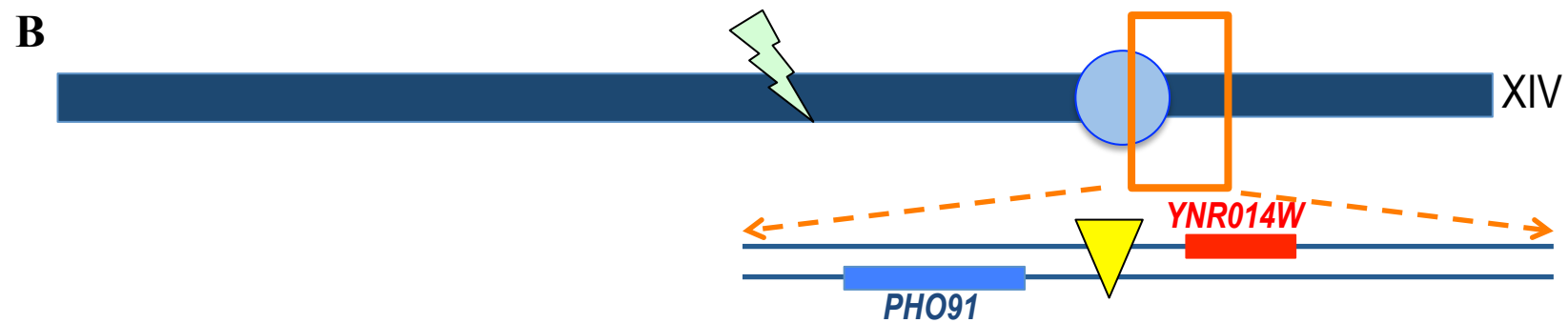

Supplement: Supplementary file 5 [file image_2.pdf]

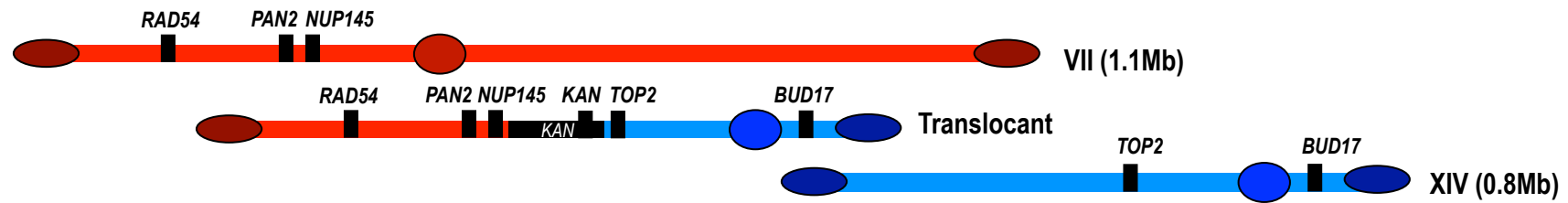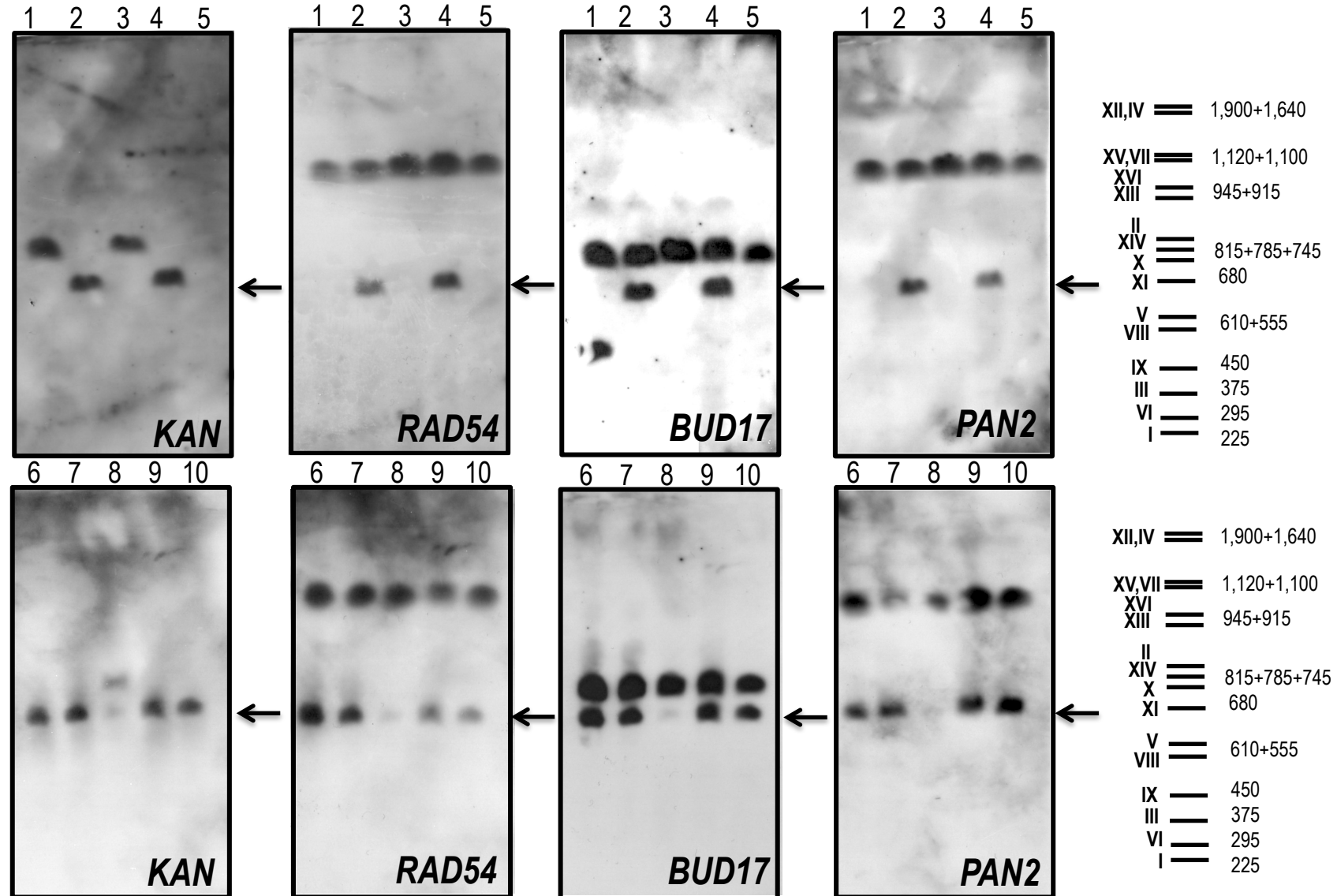

Supplement: Supplementary file 6 [file image_3.pdf]

**A**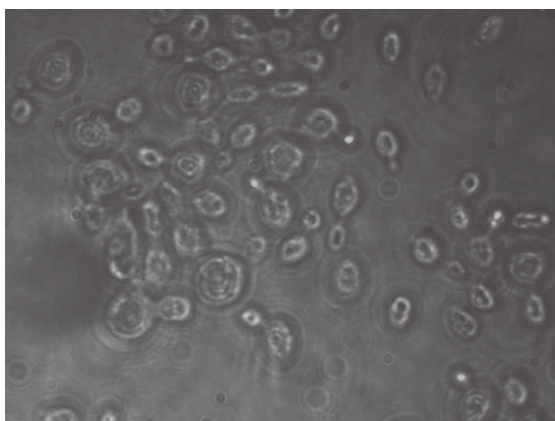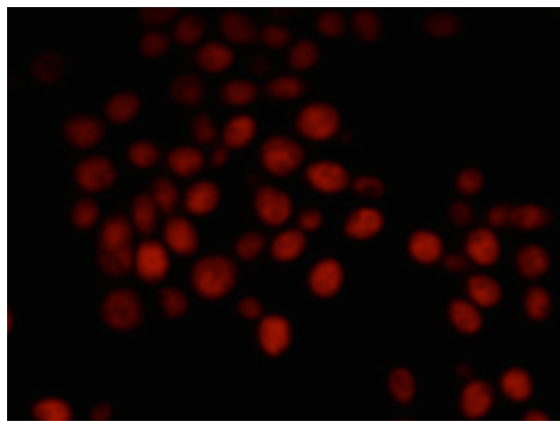**B**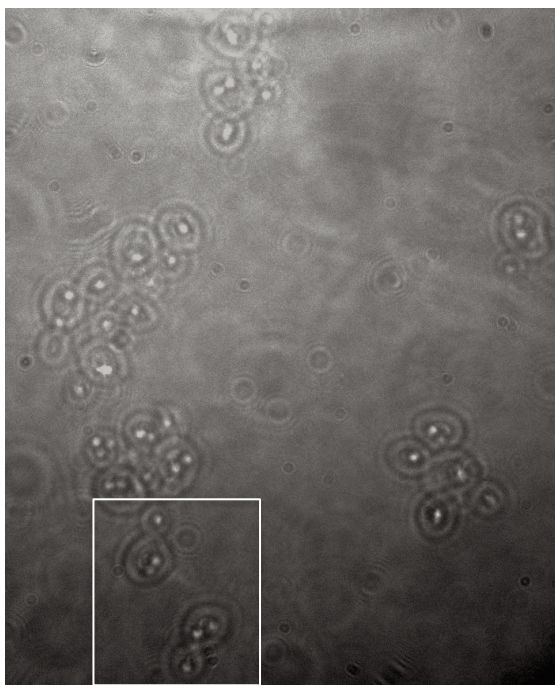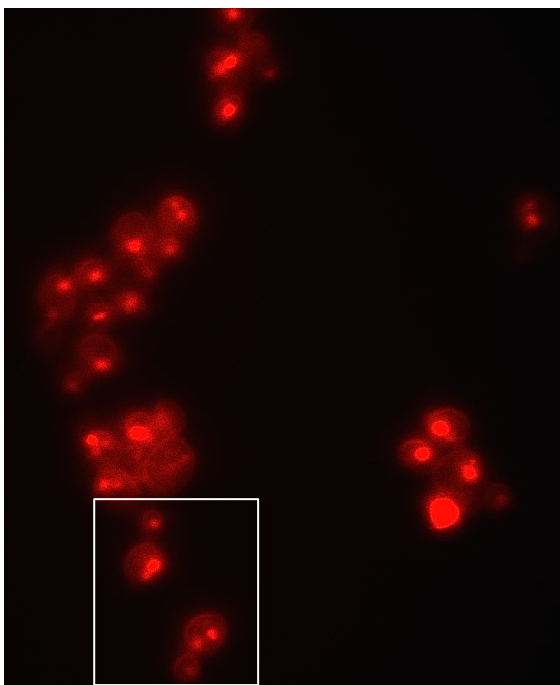**C**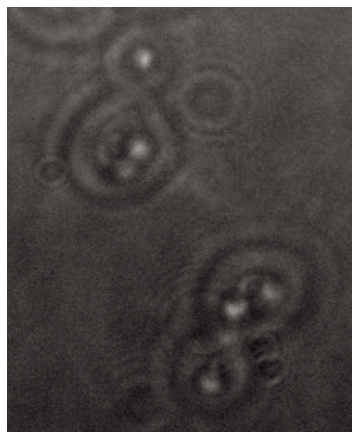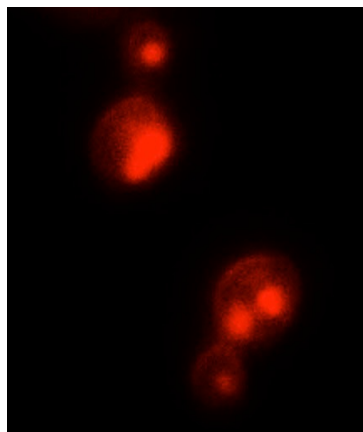

Supplement: Supplementary file 7 [file image_4.pdf]

## *NUP98* breakpoint analysis

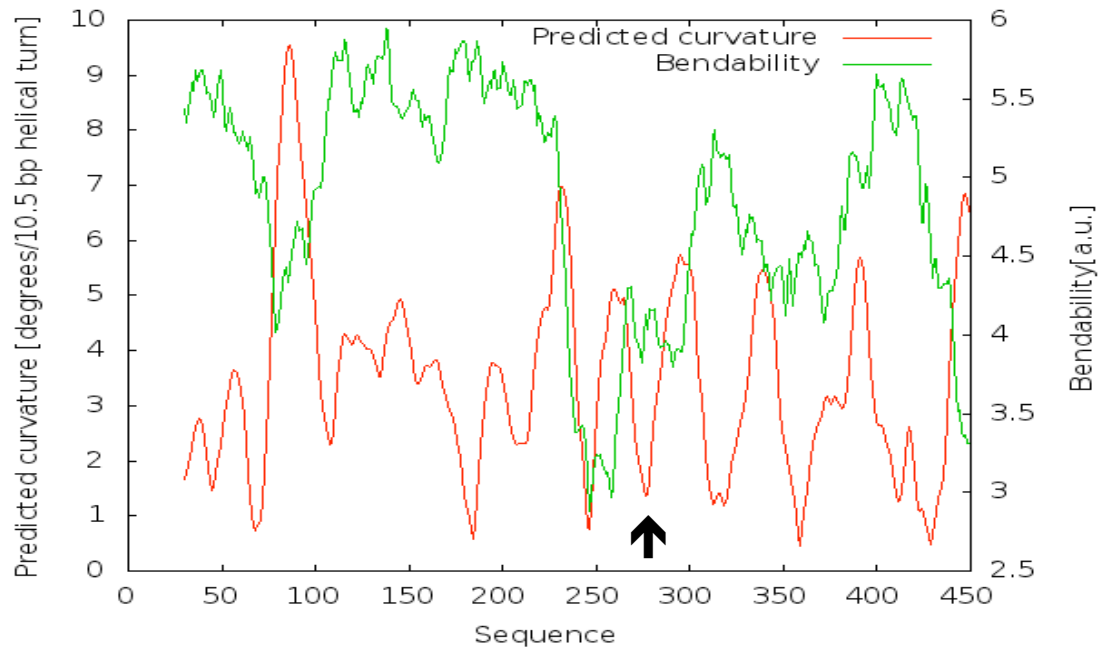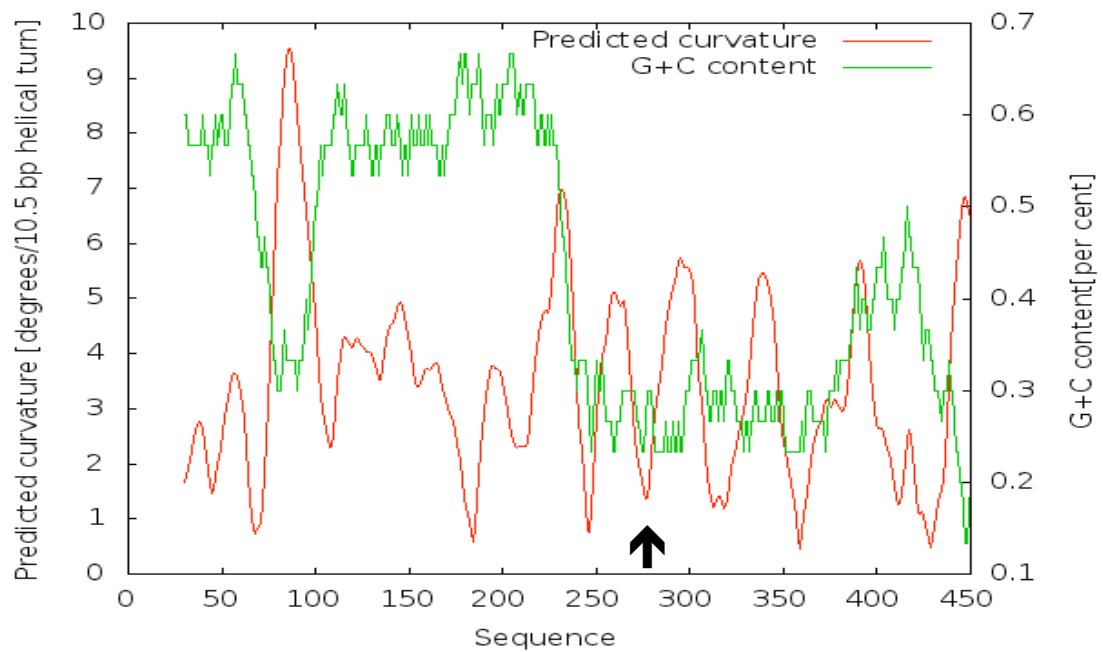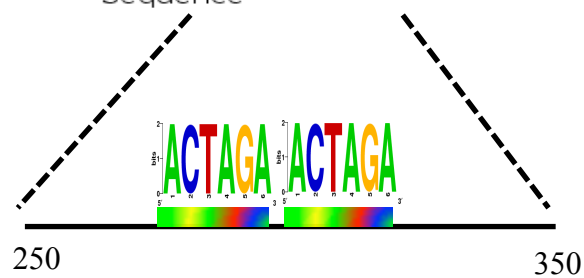

Supplement: Supplementary file 8 [file image_5.pdf]
